# Local and global microstructural and functional thalamomotor connectivity alterations in Parkinson’s disease following motor learning

**DOI:** 10.1038/s41531-026-01450-4

**Published:** 2026-06-26

**Authors:** Merav Catalogna, Efrat Sasson, Tal Tamir, Nira Saporta, Shahar Shelly, Rotem Sivan Hoffmann, Amir Amedi

**Affiliations:** 1 The Dina Recanati School of Medicine, Reichman University, Herzliya, Israel; 2 The Baruch Ivcher Institute for Brain, Cognition, and Technology, Baruch Ivcher School of Psychology, Reichman University, Herzliya, Israel; 3 WiseImage, Utrecht, Netherlands; 4 Remepy Health Ltd, Ramat Gan, Israel; 5 Department of Neurology, Rambam Medical Center, Haifa, Israel; 6 Rappaport Faculty of Medicine, Technion-Israel Institute of Technology, Haifa, Israel; 7 Radiology Department, Meir Medical Center, Kfar Sava, Israel

**Keywords:** Neurology, Neuroscience

## Abstract

Diffusion tensor imaging (DTI) is a sensitive method for detecting white-matter alterations in Parkinson’s disease (PD) and is increasingly used to capture short-term learning-induced plasticity. However, evidence for such effects in PD remains limited. In this pilot study, 32 levodopa-treated patients were randomized to a three-week digital intervention or placebo app, examining motor learning neuroplasticity. The active intervention involved a digital spatial navigation protocol combining egocentric and allocentric strategies with multisensory-deprivation principles, alongside psychological and rehabilitation modules. Neuroimaging demonstrated increased fractional anisotropy in the right ventrolateral posterior (VLp) thalamus and strengthened functional connectivity between the VLp and precentral gyrus (PreCG), both preliminary associated with improvements in motor performance. Graph-theory analysis indicated increased local efficiency in PreCG and adjacent motor networks, suggesting enhanced structural integration. Collectively, these findings raise the possibility of region-specific short-term plasticity in PD, and support DTI’s sensitivity to detect clinically meaningful training-related white-matter adaptations beyond the effects of dopaminergic therapy. Replication in larger, adequately powered samples is needed to confirm these findings.

## Introduction

Parkinson’s disease (PD), the second most common neurodegenerative disorder, is characterized by progressive motor dysfunction together with broad ranges of psychological and cognitive symptoms^1^. Levodopa, the standard first-line pharmacological therapy, primarily targets motor symptoms. However, several symptoms, such as tremor, bradykinesia, rigidity, freezing of gait, and postural instability often remain inadequately controlled^2^. Optimal disease management therefore requires a multidisciplinary team approach, including multiple nonpharmacological interventions such as physiotherapy, cue-based gait training, balance and strength exercises, and occupational therapy^2–5^. Importantly, these interventions tend to be most effective when tailored to individual needs^3^. Despite their proven value, access to such therapies is often limited by geographic, financial, logistical, and public health literacy barriers^6,7^. Digital health technologies can potentially address this gap by supporting ongoing patient engagement and facilitating individualized learning processes. However, they have yet to be efficiently integrated into routine care or undergo rigorous treatment efficacy evaluations and underlying mechanistic studies^8,9^.

**Fig. 1:**
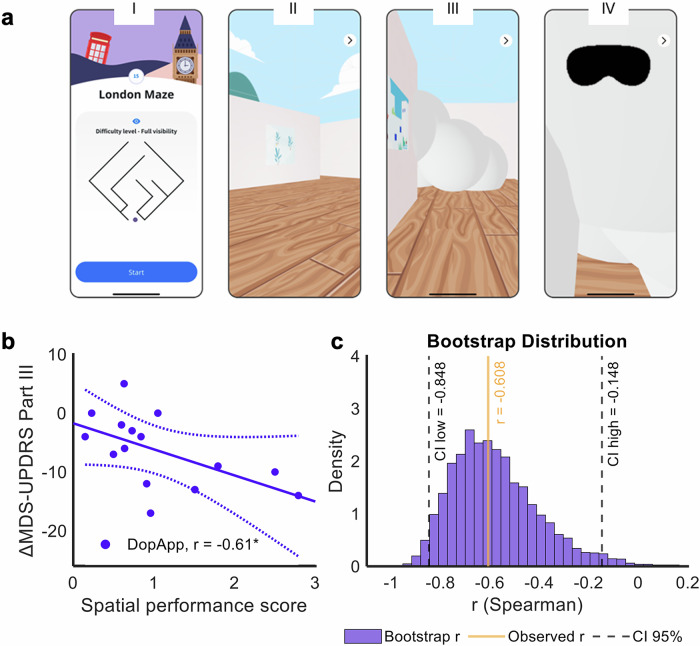
Spatial learning performance. **a** Sample sensorimotor-deprivation spatial memory screen. The exercise features virtual spatial navigation exercises that utilize digital 3D Hebb-Williams mazes and incorporate both allocentric and egocentric navigation techniques. This exercise is implemented through a progressive, three-step vision-deprivation sensorimotor training process, designed to increase navigation complexity over time. Each maze trial starts with a top-down 2D map view for allocentric navigation (left). Participants then navigate the maze fully sighted, using auditory cues for spatial information, such as distance from walls. After completing this phase, the 2D map is shown again, followed by a more challenging 3D navigation phase where 50% of the maze is masked. In the final phase, participants navigate blindfolded using only auditory feedback, employing a sensory substitution strategy. **b** A scatter plot depicting the relationship between changes in MDS-UPDRS Part III scores and spatial performance scores calculated as the number of successfully completed trials divided by the average daily usage. r, Spearman correlation value, purple dashed lines, 95% prediction bounds, *P < 0.05. **c** Bootstrap distribution of the Spearman correlation coefficient between spatial performance score and change in MDS-UPDRS Part III. The histogram represents the normalized distribution of Spearman r values obtained from 10,000 bootstrap resamples, CI, confidence intervals.

The decline in motor and cognitive functions in people with PD (PwP) is closely associated with impairments in spatial memory and navigation abilities^10^. These deficits can substantially reduce independence and negatively affect nearly all daily living activities^11,12^. Digital navigation tasks requiring fine motor control, sensorimotor integration, and visuomotor coordination offer a sensitive framework for assessing these impairments, and engaging in these activities may support voluntary movement and improve motor initiation^13^. Moreover, the dual cognitive-motor nature of these abilities requires both motor speed and step initiation, which in turn may enhance gait control. A recent intervention has introduced a unique multisensory-deprivation digital navigation exercise that involves learning novel spatial cognition and spatial memory skills through combined egocentric and allocentric navigation training^14^. This intervention is based on established neuroscience principles that have been shown to promote perceptual, cognitive, and motor learning and induce neuroplasticity across distributed sensory, motor, and cognitive brain networks^14–16^. The intervention combines multisensory integration by simultaneously engaging multiple sensory modalities (visual, auditory, and tactile), a strategy shown to enhance learning efficiency and to promote broader and higher-order neural connectivity compared with unisensory training^17–20^. Sensory substitution is also employed^21,22^, whereby a distance-to-sound algorithm conveys spatial information that is typically acquired through vision, but in this case is encoded and learned via auditory cues and spatial memory processes^15,22–24^. Additionally, gradual sensory deprivation was implemented by progressively reducing visual input from full vision to partial masking and ultimately to blindfolding. This process progressively increases reliance on non-visual, primarily auditory cues during training (Fig. 1a; Methods)^14^. Given that the visual cortex has one of the highest metabolic capacities in the human brain^25^, this controlled visual deprivation is suggested to promote the reallocation of visual neural resources toward other sensory modalities, thereby strengthening imbalanced neural networks^16,18,26^. Previous neuroimaging studies have shown that learning new motor skills, such as spatial navigation training, playing piano sequences, or finger-tapping with the non-dominant hand, induces rapid microstructural brain changes in both rodent models and healthy individuals^27–31^. Building on these findings, we investigated the neural mechanisms underlying engagement with our training program using diffusion tensor imaging (DTI).

In recent years, DTI has emerged as a valuable neuroimaging modality and a potential biomarker for assessing PD in early diagnosis, monitoring disease progression, and evaluating treatment effects^32,33^. DTI is an MRI imaging modality that quantifies the translational displacement of water molecules within tissue microenvironments^34^, typically on the order of a few micrometers, enabling the construction of a diffusion tensor, a mathematical representation of water diffusion directionality^35^. This method is highly sensitive to both the magnitude and directionality of diffusion, allowing the detection of microstructural changes triggered by transient cellular or physiological states, behavioral learning, or long-term disease progressive processes, alterations that may persist across broad timescales^36^. This tensor provides the basis for calculating quantitative metrics reflecting tissue geometric organization, for example, the mean diffusivity (MD), which reflects changes in the extracellular matrix, and fractional anisotropy (FA), which indicates the degree of fiber directionality, and fiber-tract architectures (tractography)^37^. Experimental evidence suggests that such alterations, which can occur over relatively short timescales, are driven by astrocytic remodeling, increased expression of synaptic-related proteins, and increased myelination, which may reflect underlying cellular mechanisms of neuroplasticity aimed at optimizing neural signaling efficiency^28,30,38,39^.

The pathophysiology of PD motor symptoms primarily involves dopaminergic neuron loss in the substantia nigra, leading to dysfunction within basal ganglia circuits due to neurotransmitter imbalance and progressive neurodegeneration^40^. A recent large-scale DTI study examined white matter (WM) microstructural alterations associated with disease stage and symptom severity^32^. The findings revealed widespread regional reductions in FA and increases in MD, within the cerebello-thalamo-cortical and basal ganglia–cortical loops, which became more extensive with disease progression and were associated with both motor and non-motor clinical dysfunction. Interestingly, in early-stage PD, the opposite pattern (higher FA and lower MD) was observed, suggesting early compensatory microstructural alterations^32,41^. Furthermore, PD is increasingly recognized as a network-disconnection syndrome, in which disrupted connectivity across widespread brain networks contributes to heterogeneous clinical manifestations, beyond localized brain alterations^42^. DTI–based tractography combined with graph-theory analysis can capture such disconnections through characterization of large-scale structural network organization using global and local topological metrics such as efficiency, path length, and centrality^43^. In PD, these network properties show progressive alterations, reflecting a shift from focal disruptions toward more global network disintegration and compensatory reorganization^44^. These findings suggest that graph-theory measures are promising biomarkers for evaluating PD interventions.

In the present study, we tested the hypothesis that a short-term, sensorimotor-deprivation training protocol embedded within DopApp™ (Remepy Inc., New York, NY, USA), a comprehensive mobile intervention designed to augment standard dopaminergic therapy, can enhance motor function in PD by promoting learning-induced neuroplasticity within motor networks. This study builds on a previously reported pilot investigation in the same cohort^45^, which demonstrated clinical, psychological, and resting state functional connectivity (rsFC) improvements following DopApp™ training. Extending these findings, the present work specifically tests whether DTI-based microstructural plasticity within motor networks may contribute to the underling mechanism for the observed behavioral gains. Using both voxel-based analysis (VBA) and graph theory approaches, we examined local and global microstructural changes in motor pathways and their associations with motor performance. We also hypothesized that acquiring novel spatial cognition skills enhances network efficiency and reduces path length within the basal ganglia-thalamocortical circuits, suggesting both improved network integration and underlying WM integrity properties. Finally, by combining DTI with rsFC analyses, we aimed to relate microstructural changes to functional network alterations, advancing our understanding of the biological mechanisms underlying behavioral improvements.

## Results

### Participants

All participants were drawn from the cohort described previously^45^. The DTI analysis included 32 participants (DopApp™ n = 18; placebo n = 14), and the rsMRI analysis included 31 participants (DopApp™ n = 17; placebo n = 14) as one participant was excluded from the fMRI analysis due to excessive, in-test, motion artifact. The mean age of participants was 65.8 ± 6.5 years, 59% were male and PD duration was 5.9 ± 3.3 years. Participant characteristics are shown in Table 1, with no significant between-group baseline differences (see **Supplementary Information** and Supplementary Fig. S1 for further information).

**Table 1:** Baseline characteristics

	DopApp™	Placebo	Total
N	18	14	32
Age, yrs	66.5(6.56)	65(6.48)	65.84(6.47)
Male sex	10(55.6%)	9(64.3%)	19(59.4%)
PD duration, years	6.11(3.2)	5.64(3.37)	5.91(3.23)
Hoehn & Yahr Stage			
0	0 (0%)	0 (0%)	0(0%)
1	8 (44%)	5 (36%)	13(41%)
2	9 (50%)	9 (64%)	18 (56%)
≥3	1(5.6%)	0 (0%)	1(3%)
MDS-UPDRS total score	36.56(12.88)	38.86(14.34)	37.56(13.36)
Presence of dyskinesia	7(38.9%)	3(21.4%)	10(31.2%)
Duration of levodopa treatment, years/months	3.22(1.59)	2.43(1.79)	2.88(1.7)
Daily levodopa dose, mg (range)	460.11(283.7)	475.36(238.35)	466.78(260.81)
LEDD (range)	629.56(296.53)	725.21(345.65)	671.41(317.24)
Antiparkinsonian medication use			
Dopamine agonists	5(27.8%)	5(35.7%)	10(31.2%)
MAO-B inhibitors	11(61.1%)	12(85.7%)	23(71.9%)
Amantadine	7(38.9%)	5(35.7%)	12(37.5%)
Exercise - Days per week	5.22(1.99)	4.86(2.14)	5.06(2.03)
Exercise - Minutes per day	67.5(39.9)	65.36(34.61)	66.56(37.1)
Questionnaires baseline			
MoCA	27.17(2.31)	25.86(1.75)	26.59(2.15)
PDQ39	21.19(12.21)	24.58(14.63)	22.67(13.21)
BDI-II	9.5(7.24)	9.57(7.14)	9.53(7.08)

Data are mean (SD) or *n* (%). *LEDD* levodopa equivalent daily dose, *MAO-B*, monoamine oxidase B, MoCA, Montreal Cognitive Assessment; *PDQ39* Parkinson’s Disease Questionnaire-39, *BDI-II* Beck’s Depression Inventory-II.

### Spatial learning

The spatial performance score was defined as the final level achieved at the end of the study, divided by average daily engagement time (see Methods for details). Three participants did not complete the practice level and were therefore excluded from the analysis. Among the remaining participants, the mean daily engagement time with the multisensory-deprivation navigation task was 21.4 ± 9.2 minutes/day, for 18.9 ± 4.2 days out of 21 days study duration (the daily engagement time with fine motor activities was 24.9 ± 9.9 minutes/day). The spatial performance score (1.05 ± 0.77) was not normally distributed (W = 0.867, *p* = 0.032), while the change in MDS-UPDRS Part III met the normality assumption (W = 0.983, *p* = 0.985). Given that, Spearman correlation was used, revealing a significant negative association (r = −0.61, *p* = 0.016; Fig. 1b). Bootstrap analysis yielded a 95% confidence interval of [−0.85, −0.15] (Fig. 1c). Although the interval is relatively wide, as expected for a small sample size, it consistently indicates that higher spatial performance is associated with greater motor improvement, while this interval range consistent with the expected direction of the effect. A linear regression model further supported this relationship after controlling for baseline MDS-UPDRS Part III (β₂ = −4.40, SE = 1.86, *p* = 0.036). The model explained 76% of the variance in post-intervention MDS-UPDRS Part III scores (F(2,12) = 25.08, *p* = 0.0001, R² = 0.81), suggesting that spatial performance is associated with motor outcomes independently of baseline levels (see Supplementary Table S1).

### Voxel-based analysis results

**Fig. 2:**
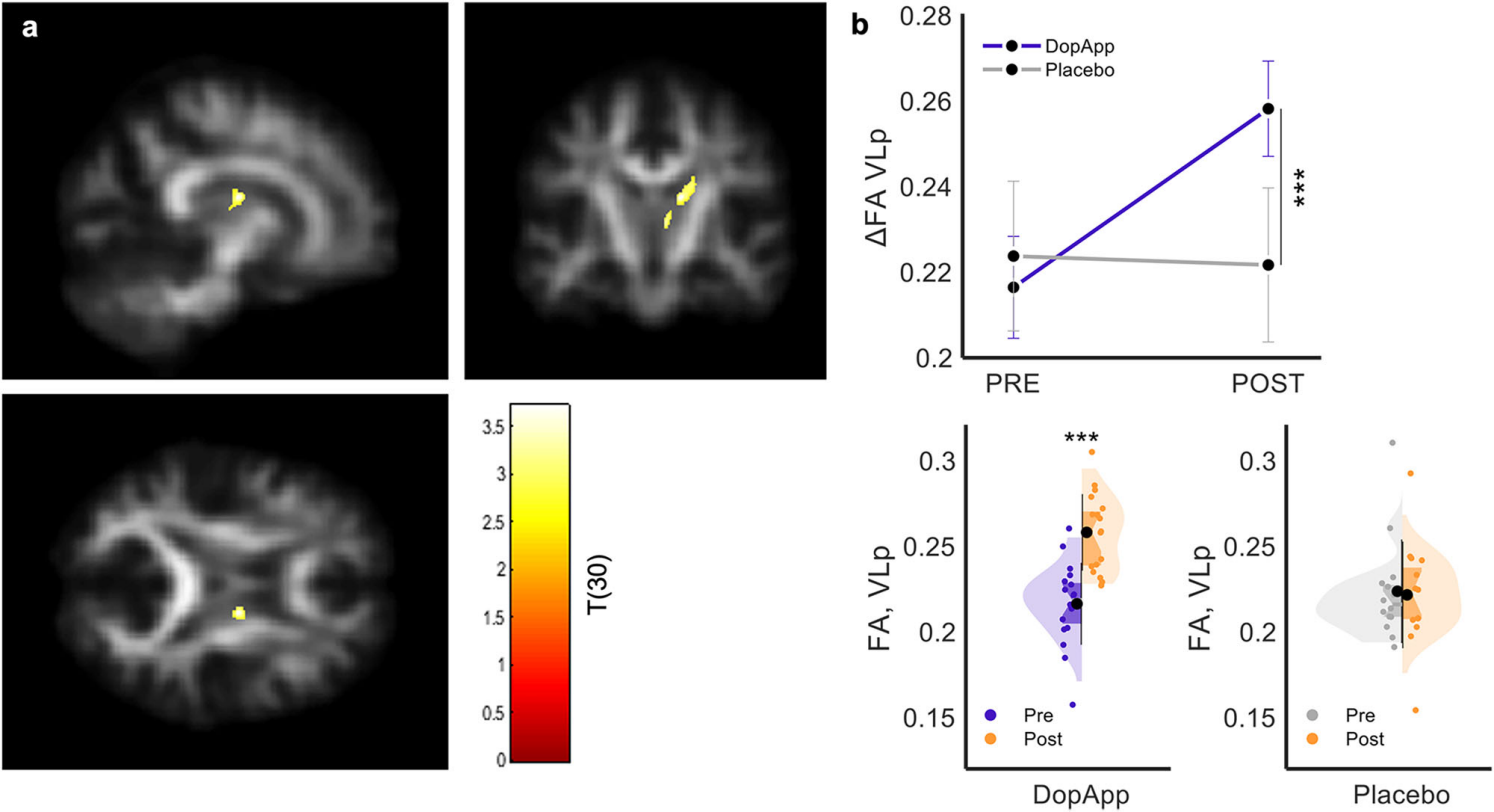
Voxel-based analysis of DTI results. **a** Group-by-time interaction ANOVA model (DopApp™ > placebo, post > pre) showing increased fractional anisotropy (FA) in the thalamo-caudate area, (k = 664, T = 3.71, *p* < 0.05, FDR corrected, small volume correction). **b** The FA cluster peak is located in the right ventral lateral posterior (VLp) thalamic subregion (+14, -10, +15). Peak group-by-time analysis results are shown in violin plots of actual distribution, and in boxplots; *** *p* < 0.001.

The whole brain group-by-time VBA model demonstrated significant fractional anisotropy (FA) increases in the trial group compared to placebo in one cluster located in the thalamus-caudate region (see Table 2 for details**)**. Cluster peak was located in the right ventral lateral posterior (VLp) thalamic subregion (k = 664, T = 3.71, *p* < 0.05, FDR corrected, small volume correction, Fig. 2). No significant results were found for MD, RD, and AD measures.

**Table 2:** Seed-based DTI group-by-time interaction analysis

Region**	R\L	X	Y	Z	Peak T	p (UNC)	p (FDR)*	K
Thalamic Nuclei								
VLp	R	14	−10	15	3.71	0.0002	0.0383	664
LD	R	6	−11	2	3.30	0.0008		
MDm	R	9	−15	9	3.25	0.0010		
Caud	R	18	−12	20	3.14	0.0013		

*VLp* ventral lateral posterior, *LD* laterodorsal, *MDm* mediodorsomedial magnocellular, *Caud*: caudate. *small volume correction, **according to the Human Connectome Project-MultiModal Parcellation Atlas (HCPex)^82^.

**Fig. 3:**
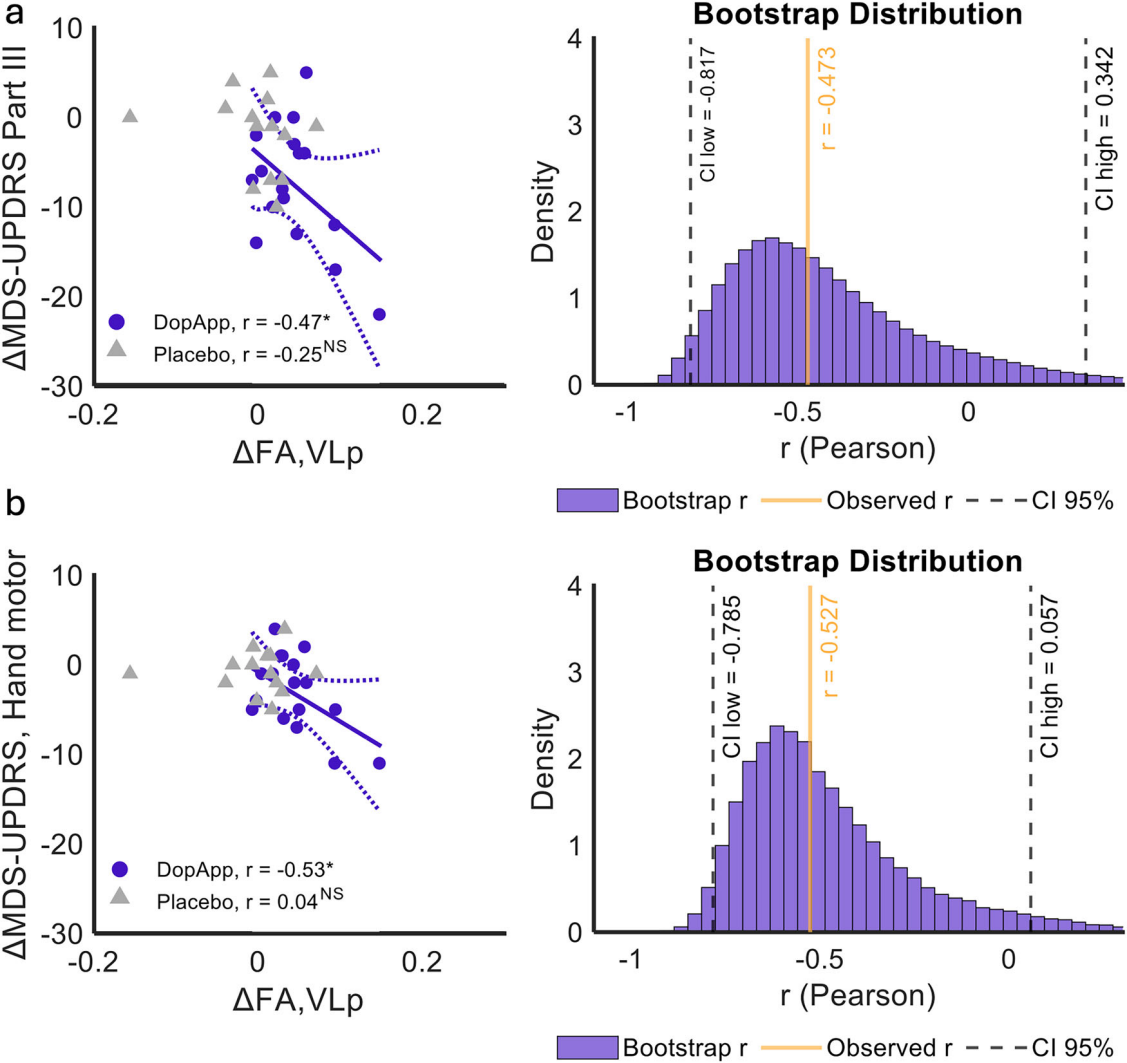
Correlations between FA alterations and clinical measures. **a** A scatter plot depicting the relationship between fractional anisotropy (FA) changes in the right ventral lateral posterior (VLp) thalamic subregion and improvement in MDS-UPDRS Part III. The bootstrap distribution shows the Pearson correlation coefficient between the spatial performance score and change in MDS-UPDRS Part III. The histogram represents the normalized distribution of Pearson r values obtained from 10,000 bootstrap resamples. **b** The same analysis for the MDS-UPDRS-based composite fine hand motor score (see Methods and **Supplementary Information** for definitions). No significant associations were found in the placebo group. r, Pearson correlations value, purple dashed lines, 95% prediction bounds, CI, confidence intervals, *p < 0.05.

In line with the peak group-by-time result, we explored the association between increased FA in the right VLp and MDS-UPDRS Part III improvements. Both variables met the normality assumption (W = 0.904, *p* = 0.075, W = 0.98, *p* = 0.996 respectively). All data points fall within ±3 standard deviations of the mean and are therefore not considered statistical outliers. Given that, Pearson correlation was used, revealing a significant negative association (DopApp™: r = −0.47, *p* = 0.047, placebo: N.S). Bootstrap analysis yielded a 95% confidence interval of [−0.817, 0.342], with a rightward skew (skewness = 1.087) that reflects uncertain statistical significance at the 95% confidence level (Fig. 3a).

A linear regression model examining the relationship between FA alterations and motor outcomes while accounting for baseline severity and group membership (F(4,26) = 34.4, *p* = 3.14 × 10^−^^10^, R² = 0.836), showed that in the DopApp™ group, greater increases in FA were significantly associated with lower follow-up MDS-UPDRS Part III scores (β_2_ = −3.967, *p* = 0.028). Since group interaction was not significant (β_4_ = −1.975, *p* = 0.214), this effect should be examined in larger studies (See Supplementary Table S2).

Focusing specifically on a composite fine hand motor score (W = 0.963, *p* = 0.653), derived from relevant MDS-UPDRS items (see Methods), greater upper-limb improvement was associated with increased FA in the right VLp (DopApp™: r = -0.53, *p* = 0.025, placebo: N.S). The corresponding 95% bootstrap confidence interval was [−0.785, 0.056], with a predominantly negative effect that marginally included zero. This pattern is consistent with a one-sided hypothesis of expected negative effects, although it should be interpreted with caution given the small sample size (Fig. 3b).

An analogous regression model was likewise significant (F(4,26) = 15.3, *p* = 1.18 × 10^-6^, R² = 0.694), showing that within the DopApp™ group, greater increases in FA were significantly associated with lower follow-up MDS-UPDRS fine hand motor scores (β_2_ = −2.682, *p* = 0.011). Here, group interaction approached significance (β_4_ = 1.707, *p* = 0.072), suggesting that this effect should be further explored in larger studies (See Supplementary Table S3).

### Thalamomotor circuit functional connectivity enhancement

**Fig. 4:**
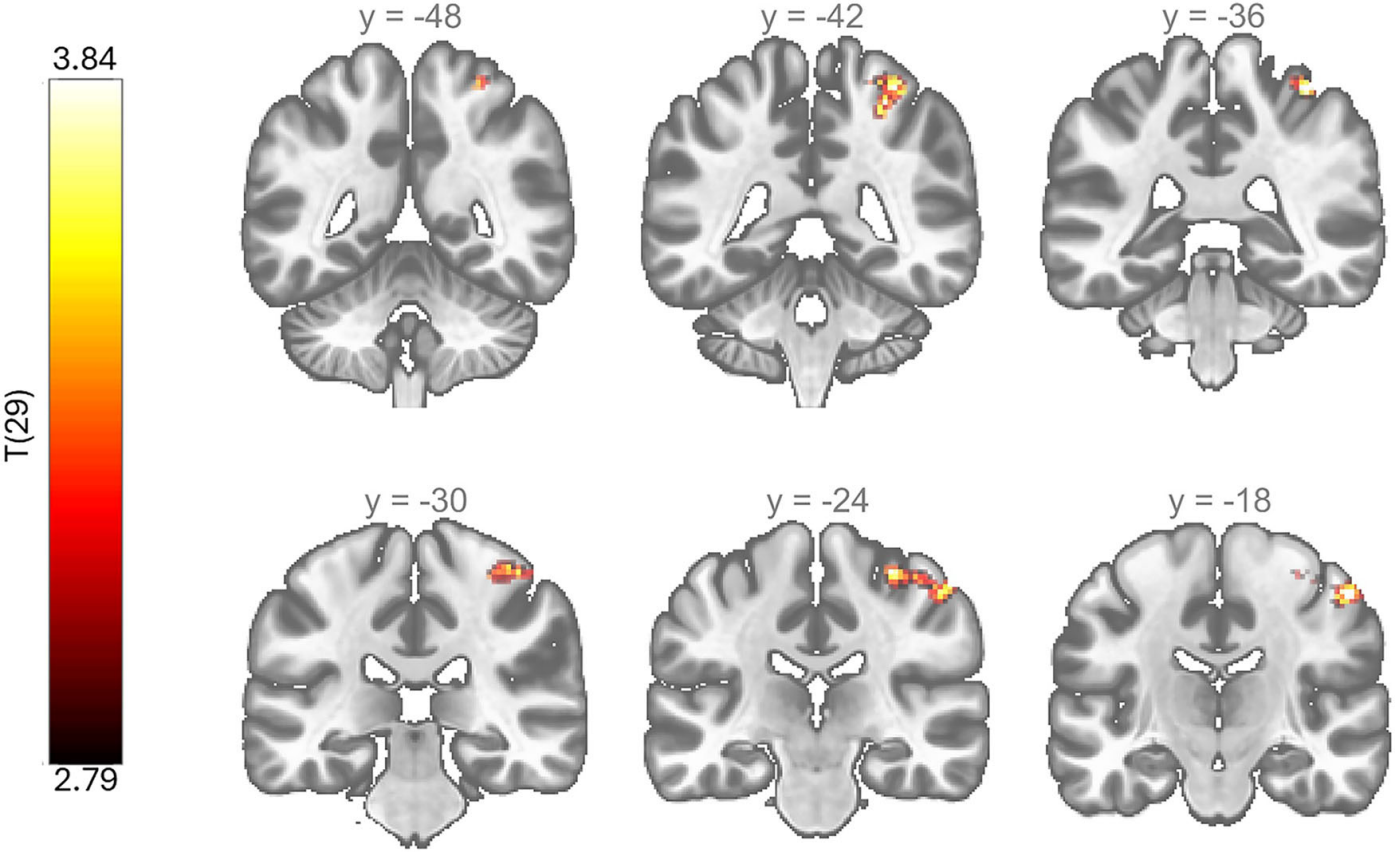
Enhancement in the thalamomotor cortex circuit functional connectivity. Whole brain seed-to-voxel, group-by-time interaction ANOVA model analysis showing significantly increased rsFC between the FA analysis cluster-based seed centered at the right VLp thalamus subregion and the motor regions in the DopApp™ group compared to placebo (DopApp™ > placebo, post > pre, voxel level *P* < 0.001, cluster level *P* < 0.05, FDR corrected). VLp: ventral lateral posterior, PreCG: precentral gyrus.

We used the previous FA cluster-peak surrounding analysis (see Methods) as a seed region for whole-brain seed-to-voxel-based rsFC analysis, to investigate possible associated functional interactions in the network level. This analysis revealed a significantly increased rsFC in the DopApp™ group compared to the placebo in the thalamomotor circuit, as shown in Fig. 4. A significant group-by-time interaction was demonstrated in a main cluster covering the following regions: right postcentral gyrus (PostCG, k = 350, T = 3.31, peak, +44, -26, +56), right superior parietal lobule (SPL, k = 164, T = 3.26, peak, +32, -42, +58) and the right precentral gyrus (PreCG, k = 121, T = 3.11, peak, +36, -20, +58).

Next, we explored the association between rsFC alterations and MDS-UPDRS-III improvements. Both change in VLp-PreCG rsFC data and change in MDS-UPDRS Part III met the normality assumption (W = 0.97, *p* = 0.799, W = 0.98, *p* = 0.95 respectively). All data points fall within ±3 standard deviations of the mean and are therefore not considered statistical outliers. Given that, Pearson correlation was used, revealing a significant negative association (r = −0.553, *p* = 0.027, placebo: N.S). Bootstrap analysis yielded a 95% confidence interval of [−0.811, 0.003] (Supplementary Fig. S2). The effect was predominantly negative, with the interval marginally including zero. This pattern is consistent with a one-sided hypothesis of expected negative effects, although it should be interpreted with caution given the small sample size. A linear regression model assessing the relationship between rsFC changes and motor outcomes (controlling for baseline severity and group) was significant (F(4,26) = 28.4, *p* = 3.72 × 10⁻⁹, R² = 0.814). Group membership was a significant predictor (β = −6.39, *p* = 0.016), indicating that the DopApp™ group had ~6.4-point lower MDS-UPDRS III scores at follow-up compared with controls after adjustment, consistent with a clinically meaningful treatment effect. However, regression modeling did not show significant results regarding the rsFC effect, possibly reflecting limited statistical power given the sample size (see Supplementary Table S4).

### Post-hoc analysis: Network topology analysis results

**Fig. 5:**
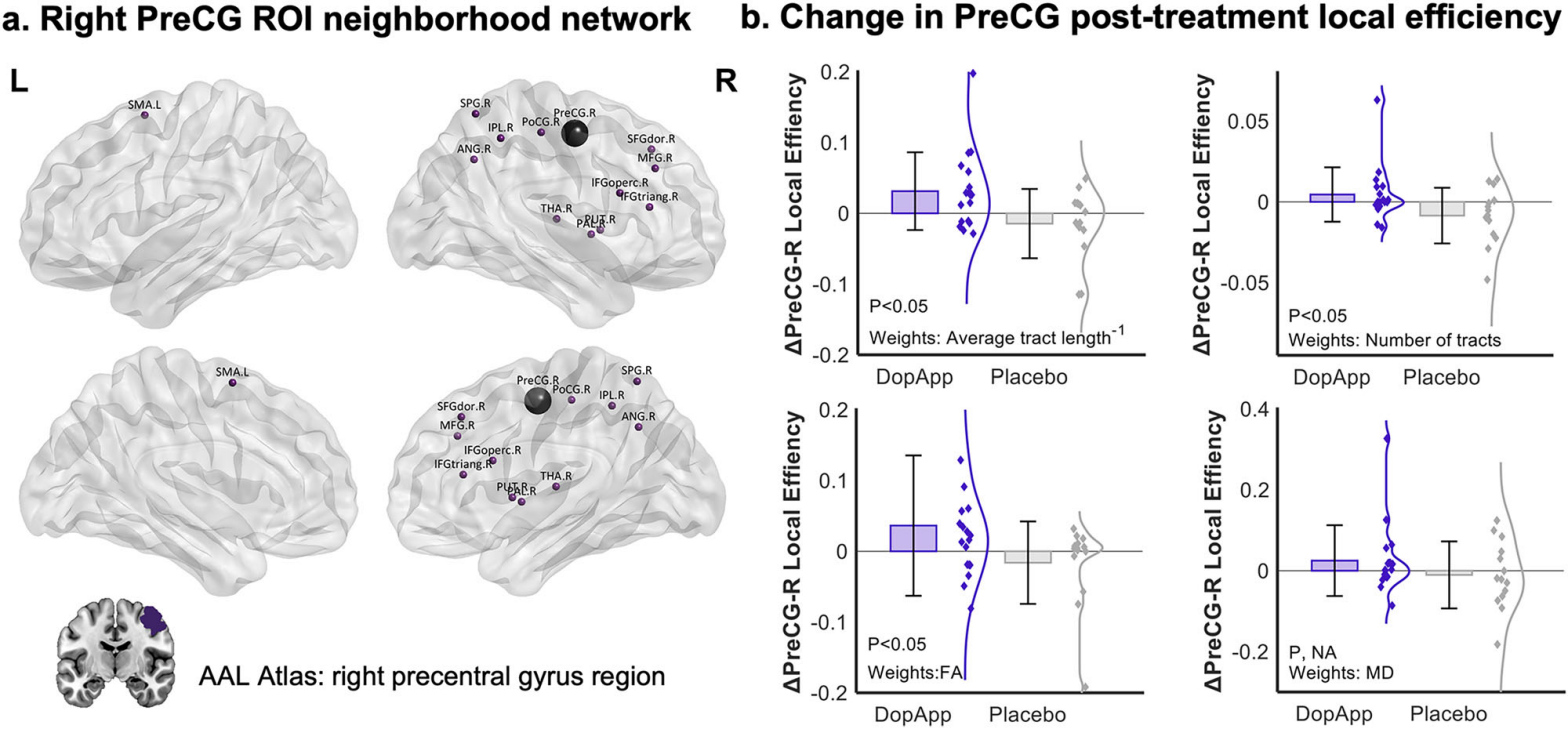
Network topology analysis results. **a** The right PreCG ROI and its topological neighboring brain regions, defined as adjusted nodes directly connected by a non-zero edge above the FA-threshold. This sub-network (subgraph) demonstrates coupling with premotor cortex, basal ganglia and posterior parietal cortex regions. Brain images were created using BrainNet Viewer software (http://www.nitrc.org/projects/bnv/). **b** Changes in PreCG post-treatment local efficiency for different edge-weight matrices. Significant group-by-time results were found for average tract lenth^−^^1^ and FA edge-weights, suggesting improved architecture of structural wiring and enhanced WM integrity within motor-related pathways. FA, fractional anisotropy, MD, mean diffusivity, AAL, Automated Anatomical Labeling Atlas^77^, PreCG, precentral gyrus. P-value, group-by-time ANOVA model.

In terms of global topology measures, global network measures were comparable at baseline, and no significant group-by-time differences were detected following intervention (Supplementary Table S5). Therefore, network post-hoc analysis at the regional level, was performed in the PreCG node and in the topologically neighboring brain regions, defined as adjusted nodes directly connected by a non-zero edge above the FA-threshold (see Methods). A significant increase in local efficiency was found in the right PreCG node and its neighboring brain regions, encompassing basal ganglia, motor, and parietal regions that collectively support motor execution and coordination (Fig. 5a). These increases were most pronounced when using average tract length^-1^ (d = 0.874, F = 6.01, *p* < 0.02, Mann-Whitney U test, *p* < 0.04) and FA-based (d = 0.755, F = 4.49, *p* < 0.05, Mann-Whitney U test, *p* < 0.04) edge-cost measures, suggesting improved architecture of structural wiring and enhanced WM integrity within the motor-related pathways. When the number of tracts was used as the edge-cost measure, significant effects were initially observed (Fig. 5b). However, these effects did not survive exclusion of the outliers. Accordingly, no robust between-group differences were detected, suggesting that the observed effects were not driven by increased node strength (i.e., higher streamline counts), but rather by enhanced efficiency and quality of existing structural connections. Taken together with the lack of changes in global efficiency, these results suggest that the intervention selectively modulated local rather than global network properties, highlighting targeted improvements in structural connectivity within the motor system.

### Sensitivity power analysis

A post-hoc sensitivity power analysis conducted using G*Power (α = 0.05, power = 0.80) indicated that the present study is primarily powered to detect large effect sizes (Cohen’s d = 1.03), accordingly, smaller or moderate effects in FA may not have been reliably detected. Based on the observed effect sizes, and accounting for cluster-level multiple-comparison considerations, prospective power estimates suggest that approximately 32 participants per arm would be required in future studies (80% power, α = 0.005, two-sided).

## Discussion

This pilot study suggests that diffusion MRI can capture early structural plasticity in thalamomotor pathways following a comprehensive, multimodal digital intervention that integrates learning-based spatial sensorimotor-deprivation training, psychological modules, and rehabilitation exercises in levodopa-treated PwP compared to a placebo-app. Whole-brain analyses showed that the main location of brain plasticity, highlighted by increased FA in the right thalamus, was particularly within the VLp (Fig. 2), where the alterations preliminary associated with MDS-UPDRS Part III motor scores (Fig. 3). Increases in local efficiency within the right PreCG and its neighboring motor-parietal-basal ganglia network suggest improved structural wiring architecture and enhanced integrity of WM pathways supporting motor function (Fig. 5). These observations were strengthened by increased rsFC in the VLp-PreCG pathway (Fig. 4), which related to motor improvements, associating the structural adaptations to their functional and behavioral expressions and supporting the findings’ reliability and specificity. Building on previously reported clinical, psychological, and rsFC findings from this cohort, demonstrating significant motor function improvements and rsFC alterations in the thalamomotor network^45^, the current results extend this evidence by revealing corresponding WM microstructural changes. Taken together, these findings demonstrate the clinical importance and effectiveness of targeting spatial cognition via multimodal sensorimotor training in PD, highlight diffusion MRI as a sensitive biomarker of rapid, training-induced neuroplasticity, and provide a potential mechanistic framework for behaviorally driven digital-pharmacological interactions^46^.

Spatial symptoms and deficits in spatial navigation abilities are common in PwP and are associated with both motor and non-motor symptoms^11,12^. In this study, the associations between enhanced performance following spatial sensorimotor-deprivation training and improvements in clinical motor function (MDS-UPDRS Part III) were supported by converging statistical approaches, suggesting that the level of performance achieved on the spatial task is meaningfully associated with clinical motor improvement independent of baseline severity. These results align with a growing body of evidence showing that short-term (single session and up to several weeks) interventional learning experiences, such as motor, spatial memory, cognitive skill learning, or psychological behavioral learning, can induce rapid microstructural plasticity within relevant neural pathways^29–31,47,48^. The present findings extend this literature by demonstrating short-term neuroplasticity in PwP by linking these structural alterations to clinically meaningful gains in motor function. From a clinical perspective, these results highlight the importance of evaluating treatment effect durability and determining whether continued or periodic maintenance training is necessary to preserve functional improvements and support long-term clinical outcomes in routine PD care. Importantly, navigational abilities are known to decline with normal aging^49–51^ and are further impaired in pathological conditions, such as mild cognitive impairment (MCI) and dementia^52–54^, and Alzheimer’s disease (AD)^55,56^, making this strategy valuable for broader cognitive and functional benefits across aging and neurodegenerative disorders.

The thalamus serves as a key integrative hub within the basal ganglia–thalamocortical circuits that regulate motor, cognitive, and limbic functions^57^. Neurodegeneration affecting these pathways contributes to the core motor and non-motor symptoms of PD^58–60^. More specifically, the ventral lateral (VL) thalamic nuclei integrate motor control by receiving afferent inputs from the basal ganglia output nuclei and cerebellum, and projecting primarily to the primary motor cortex (PreCG), premotor (PMC) and supplementary motor areas (SMA)^61^. These nuclei have been implicated in modulating PD motor symptoms^62,63^. In this study, we demonstrate increased FA within the right thalamus, most prominently in the VLp subregion, which directly projects to the PreCG. This increase was significantly correlated with MDS-UPDRS Part III motor scores and the MDS-UPDRS-based composite fine hand motor score, supporting an association with fine motor performance. Importantly, we also observed that increased functional connectivity between the VLp and PreCG was associated with greater motor improvement (MDS-UPDRS Part III), suggesting a functional relevance of the structural changes identified in this region.

Previous studies have reported decreased thalamic FA in PwP compared to healthy controls, across different disease stages and symptom profiles. For example, reduced FA in the right thalamus has been observed in both de novo patients^64^ and medicated patients in the OFF state^65^ with comparable motor severity (MDS-UPDRS Part III, Hoehn & Yahr). In another study in medicated patients exhibiting persistent gait impairments, higher thalamic FA values correlated with better gait performance in the ON state^66^. Conversely, decreased thalamic FA correlated negatively with motor speed and balance in postural instability gait difficulty (PIGD)^67^. Furthermore, thalamic diffusion properties have been proposed as markers to distinguish tremor-dominant from postural/gait-dominant PD phenotypes^68,69^. Taken together, our results suggest that coordinated microstructural and functional plasticity within the VL thalamic nuclei may support motor improvements in PwP, highlighting this region as a potential target for therapeutic interventions aimed at modulating motor function.

In addition to examining local microstructural changes, we used graph-theory analysis based on tractography to characterize the brain’s global and regional structural networks. Increased local efficiency was detected in the right PreCG and its neighboring brain regions, including basal ganglia, motor, and parietal regions implicated in motor execution and in coordination. Local efficiency is used to measure the ability of information transmission. Therefore, increases may indicate higher abilities of neural signaling that may reflect learning-related plasticity^43^. Additionally, these increases were related to average tract length and FA-based edge-cost measures, suggesting improved structural wiring and enhanced integrity of motor function WM pathways. By recognizing PD as a network-disconnection syndrome^42^, these findings suggest compensatory reorganization at the network level beyond localized changes. However, contrary to our initial hypothesis, shaped by prior work comparing PwP to health controls^44^, we did not observe post-training changes in global measures (global efficiency, density, global path length, etc.). A possible explanation is that alterations in these global metrics tend to emerge gradually over different disease stages and require longer timeframes to manifest^32^, whereas our intervention focused on short-term training effects.

Despite growing interest in neuroplasticity in PD, most network topology studies are cross-sectional^32^, and longitudinal investigations evaluating treatment-related changes using diffusion MRI are scarce. One notable study is an 8-week cognitive training program in PwP that induced alterations in local WM microstructure. These changes correlated with cognitive improvement, without detectable changes in global brain network topology^47^. Together, these results suggest that short, targeted interventions in PwP primarily induce localized microstructural plasticity related to functional improvement within key brain areas and their immediate neighboring regions, rather than widespread reorganization of large-scale structural networks. Given the limited duration of such interventions, these results suggest meaningful early indicators of neural adaptability, with longer or repeated training likely necessary to elicit broader network remodeling.

This study has several limitations. The small sample size, for which post-hoc power analysis indicates adequate sensitivity only for large effect sizes, may have limited statistical power to detect significant effects in both local and global measures, despite observed group-level improvements in the active arm compared with placebo. Nevertheless, the consistency and regional specificity of the effects, particularly within targeted brain areas and thalamic subregions across complementary analysis types, alongside associations with clinical motor measures, strengthen the validity of our results as a framework for future studies. An additional limitation is that the spatial performance score employed captures overall engagement and progression but does not fully reflect qualitative aspects of learning, such as error patterns, strategy use, or reaction time. Future studies should incorporate more refined behavioral measures to better characterize learning dynamics. A further limitation is the comprehensive, multimodal nature of the DopApp™ intervention, which combined spatial navigation training, psychological modules, and simple sensorimotor (tapping-based) exercises within an integrated digital platform. This design reflects a realistic, ecologically valid digital therapeutic approach, but limits the ability to isolate the contribution of individual components to the observed effects. While this task is theoretically distinct from simple sensorimotor exercises, as it engages higher-order spatial planning and continuous sensorimotor deprivation, the current study design does not allow attribution of the observed behavioral or neural effects to specific modules, nor to their interaction. Future studies should therefore employ mechanistic, component-level designs, such as dismantling studies that selectively remove individual modules, or factorial designs that independently manipulate spatial, cognitive, and motor components. Such approaches would help identify the most effective elements and their interactions, ultimately informing the development of more targeted, patient-specific, and clinically optimized digital interventions. Finally, the placebo app did not include motor tasks, which may limit comparisons regarding motor-related outcomes. To confirm and extend these neural findings, larger multicenter studies employing standardized imaging protocols and a follow-up period are needed.

In conclusion, these findings demonstrate that multimodal digital interventions including a novel multisensory-deprivation motor task, combined with dopaminergic therapy, can enhance neuroplastic mechanisms within thalamocortical motor circuits in PwP. By engaging multisensory integration across visual and auditory modalities, utilizing sensory substitution through spatial auditory cues, and incorporating gradual visual deprivation, this intervention may induce microstructural and functional reorganization within neural networks. Such neuroplastic adaptations are hypothesized to reflect improvements in axonal integrity and network efficiency, though further investigation is needed to elucidate the underlying mechanisms. This study complements previous research on microstructural effects targeted by short-term leaning process and supports the potential of combined digital and pharmacological approaches to induce adaptive plasticity, offering promising avenues for personalized rehabilitation and improved clinical outcomes in PwP.

## Methods

### Study design and participants

This pilot study builds on a previously reported pilot investigation in the same cohort^45^. Briefly, the study was conducted at the Baruch Ivcher Institute for Brain, Cognition & Technology (BCT) within the School of Psychology at Reichman University, Israel, between August 11^th^ 2024, and October 23^rd^, 2024. Eligible participants, aged 45-76 years old with a recorded PD diagnosis and treated with stable daily regimens of levodopa (stable regimen of 150-1500 mg/day for ≥30 days with a maximum of five doses per day), were randomly allocated to either the DopApp™ or the placebo app for three weeks. Randomization was performed using a 1:1 ratio with a block size of four, via a computer-generated randomization table. PwP with additional major unstable neurologic, psychiatric or medical conditions were excluded. Further protocol details are provided in the **Supplementary information**. The study was conducted according to the Declaration of Helsinki guidelines and local institutional regulations. The study was approved by the Reichman University Institutional Review Board (IRB) (P_2024094, on April 18^th^, 2024), and the neuroimaging study protocol was approved by Meir Medical Center’s IRB (122-24-MMC, Israeli registration, MOH_2024-08-06_013567 on August 5^th^, 2024). All participants provided written informed consent prior to their inclusion.

### Treatment

The DopApp™ intervention is a comprehensive multimodal program integrating spatial-cognitive, psychological, and rehabilitation-focused exercises across well-established disciplines. It incorporates principles of multisensory (vision, audio) integration, sensory substitution, and masking. These techniques were digitized and delivered through short interactive videos, audio, and engaging games. DopApp™ interventions were structured into 21 daily routines as detailed in the **Supplementary Information** and Supplementary Fig. S3. Importantly, the spatial-cognitive component constituted the majority of the motor-active protocol **(**see the spatial navigation learning task section for details**)**.

The app utilized adaptive algorithms to dynamically adjust task difficulty based on real-time performance metrics, engagement, and progression. The placebo app had a similar appearance to the DopApp™, and onboarding steps were identical. The placebo intervention protocol consisted of a daily regimen featuring PD-related content such as nutritional guidance and the general benefits of physical activity. All study participants received daily new content, along with app usage reminders, as well as access to an on-demand library of previously completed interventions.

### Spatial navigation learning task

The virtual spatial navigation training was based on a fully digitized set of Hebb–Williams (HW) mazes^70^. The protocol integrates principles of sensory integration, sensory deprivation, and sensory substitution, creating a multimodal training environment intended to accelerate spatial learning and enhance coordination between sensory and cognitive brain networks^14–16,71^.

A central feature of the protocol is the combined use of egocentric and allocentric navigation strategies delivered through a three-step, progressively challenging blindfold-training paradigm. Each maze trial begins with the presentation of a top-view map, enabling allocentric encoding by allowing participants to form a cognitive map of the maze. This is followed by a fully sighted virtual 3D navigation phase enriched with auditory cues that provide continuous information about distance from the walls. After successfully completing this phase, participants repeat the trial under increased sensory constraints: 50% of the maze is randomly masked, reducing visual information and encouraging integration of residual visual cues with auditory feedback and spatial memory. In the final step, the top-view map is removed, and participants navigate the maze blindfolded, relying solely on spatial memory and auditory input (Fig. 1a). Auditory information is delivered through a distance-to-frequency algorithm in which higher sound frequencies indicate proximity to a wall and lower frequencies indicate greater distance. Footstep sounds signal a clear passage. Navigation is controlled via touchscreen swipes in eight 45° directions (or straight movement). The primary task objective is successful wayfinding, with participants instructed to find the fastest route to the exit while avoiding wall collisions^14^. The spatial learning performance score is calculated by dividing the number of successfully completed trials by the average daily usage (the mean time spent per day, minutes/day), which captures daily engagement or daily dose for each content type.

### Outcomes and assessments

Participants were assessed at baseline and following a 3-week intervention period using the MDS-UPDRS scale^72^, and standardized PD clinical and psychological questionnaires. MDS-UPDRS Part III assessments were conducted in the ON state as determined by the patient’s subjective experience of improvement in motor function after taking the medication. A composite fine hand motor score was used as an outcome measure, derived from selected MDS-UPDRS items related to fine hand motor activities in daily living, tremors, and finger tapping (see **Supplementary Information**). This score was designed to focus exclusively on behavioral changes, most relevant to the motor domain targeted by the app intervention, rather than overall motor severity.

In this study, we focused on neuroimaging aspects of motor symptoms and their potential associations with DTI and rsFC alterations. This study represents a secondary neuroimaging analysis of the DopApp™ trial. The trial design, intervention protocol, clinical assessments, psychological measures, and resting-state fMRI analyses were previously described in detail^45^. See also additional details in the **Supplementary Information**.

### Brain imaging

Brain imaging MRI scans were performed on a MAGNETOM Prisma 3 T Scanner, configured with 64-channel receiver head coils (Siemens Healthcare, Erlangen, Germany), at the Ruth and Meir Rosental Brain Imaging Center (MRI), Reichman University. Due to dopaminergic effects on rsFC^73^, PD medications were administered two hours prior to MRI scans. The MRI protocol included the following sequences:

1. Two runs of rs-fMRI scans (300 volumes, 9:28 min each) were acquired using a multiband echo planar imaging sequence, CMRR EPI 2D^74,75^. Scan parameters: TR: 1870 ms, TE: 30 ms, flip angle: 75°, voxel size: 3.0 × 3.0 × 2.0 mm, FOV: 192, number of slices: 58, axial slices parallel to the AP-PC plane. During scanning, participants were asked to remain still and relaxed, with their eyes fixated on a cross, and without deliberately thinking of anything. Foam pads and earplugs were employed to reduce head motion and scanning noise.
2. Whole brain diffusion weighted images were acquired with the following parameters: 60 axial slices, slice thickness = 2 mm, voxel size = 2 × 2 mm, TR = 3400 ms, TE = 63 ms, and matrix = 248 × 124 mm, SMS factor = 3. Diffusion gradients were applied along 64 noncollinear directions (b = 1000 s/mm^2^) and seven volumes without diffusion weighting, including five volumes in read directions and two volumes in phase direction to compensate for EPI distortions.
3. Structural T1-weighted MRI scans were acquired for co-registration purposes using a T1-weighted 3D magnetization-prepared rapid gradient-echo (MPRAGE) sequence in a sagittal plane with 1 mm isotropic resolution. Sequence parameters: TR: 2,000 ms, TE: 1.9 ms, flip angle: 9°, TI: 920 ms, FOV: 256 ×256, and 176 contiguous slices.
4. The MRI protocol also included T2-fluid-attenuated inversion recovery (FLAIR) sequences, using standard parameters for clinical brain evaluation.

### DTI data analysis

Diffusion tensor imaging (DTI) data were preprocessed using ExploreDTI software (http://www.ExploreDTI.com, version 4.8.6)^76^. Preprocessing included correction for subject motion and eddy current–induced distortions, as well as tensor regularization. High-resolution 3D T1-weighted images underwent tissue segmentation and were co-registered to the mean diffusion-weighted image (DWI). The registered 3D T1-weighted image was spatially normalized to the MNI standard space, and the same normalization parameters were then applied to the diffusion maps, including fractional anisotropy (FA), mean diffusivity (MD), axial diffusivity (AD), and radial diffusivity (RD). See Supplementary Table S6, for mathematical definitions. Normalized maps were smoothed using a Gaussian kernel of 6 mm full width at half maximum (FWHM). For subsequent analyses, a white matter mask was applied based on an FA threshold of 0.2. In addition, a non-CSF mask was derived from the gray matter (GM) and white matter (WM) segmentations to exclude CSF.

**Fig. 6:**
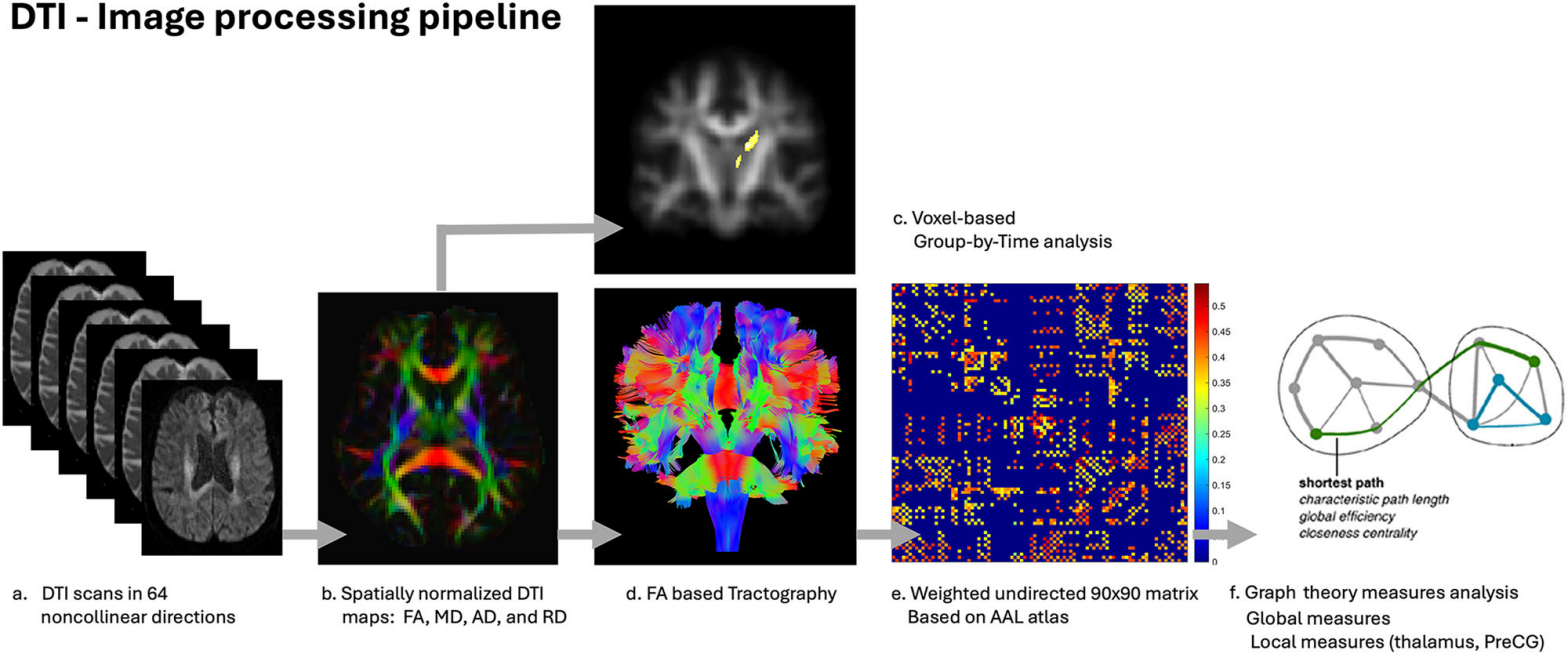
DTI image processing pipeline. **a** Diffusion-weighted imaging (DWI) scans were acquired in 64 noncollinear directions. Each of the 32 participants was scanned twice, at baseline and after the intervention. **b** Diffusion tensor imaging (DTI) maps were calculated. In the FA map, colored tensors (red/green/blue) represent the principal diffusion orientations of water molecules. **c** Voxel-based structural connectivity group analyses were performed on the quantitative maps (FA, MD, AD, RD) to test group-by-time interaction effects. **d** Tractography was performed in ExploreDTI^76^ between each inversely transformed 90 regions AAL atlas ROI pair, using an angle threshold > 45° and FA < 0.2 as criteria. **e** From these, 90 × 90 connectivity matrices (CMs) were derived for the following edge-cost measures: fractional anisotropy (FA), mean diffusivity (MD), number of tracts, average tract length, and a binary CM. **f** Graph theory measures^43^: global (density, efficiency, strength, betweenness centrality) and local (local efficiency, local strength) were calculated using the Brain Connectivity Toolbox (https://sites.google.com/site/bctnet/).

Network topology analysis: We examined weighted graph-theory measures that reflect network properties (Fig. 6). First, whole-brain structural connectivity matrices were computed using a network analysis tool implemented in the ExploreDTI software. We used the Automated Anatomical Labeling (AAL) Atlas as a template with 90 distinct regions of interest (ROIs), which were registered to each subject’s dataset^77^. Tractography was performed between every inversely transformed AAL-ROI pair using the criteria of angle threshold > 45° and FA < 0.2. Then, 90 × 90 connectivity matrices (CMs) were derived and examined according to the following variables (edge-cost measures): fractional anisotropy (FA), mean diffusion (MD), number of tracts, average tract length, and a binary CM^37,78^. Topological global (Density, Efficiency, Strength, Betweenness centrality) and local (local Efficacy, local Strength) measures (see Supplementary Table S6 for mathematical definitions) were calculated on normalized and symmetrical CMs using the brain connectivity toolbox (https://sites.google.com/site/bctnet/) based on the work of Rubinov and Sporns^43^. Local measures were specifically computed within the PreCG nodes as a post-hoc analysis following the rsFC analysis results.

### BOLD data preprocessing and seed selection

RsFC analysis was carried out using CONN (RRID: SCR_009550, https://web.conn-toolbox.org, v22a), as implemented using statistical parametric mapping software SPM12 (http://www.fil.ion.ucl.ac.uk/spm). The functional volumes preprocessing pipeline included realignment with correction of susceptibility distortion interactions, slice timing correction, outlier detection, direct segmentation, and MNI-space normalization, with a resolution voxel size of 2.0 × 2.0 × 2.0 mm, and spatial smoothing (8 mm FWHM Gaussian kernel) steps^79^. A component-based noise correction procedure (CompCor) approach^80^ was used to identify additional confounding temporal factors controlling for physiological noise, BOLD signal present in WM, and head motion effects. Finally, residual BOLD time series were then bandpass-filtered at a frequency range of 0.008–0.09 Hz. Individual connectivity maps were generated using the seed-to-voxel approach. Bivariate correlation analysis was used to determine the linear association of the BOLD time series between the seed and significant voxel clusters. Fisher’s Z transformation was applied to the correlation coefficients to satisfy normality assumptions. Finally, participants with head motions of >3 mm in any direction between volumes, rotations of >3° in any axis during scanning, or mean framewise displacement (FD) scan-to-scan head-motion > 0.5, in either the pre- or post-treatment maps, in both runs, were excluded from the dataset. The seed region mask was constructed as a 6 mm radius sphere centered on the peak coordinate of the FA cluster identified in the DTI VBA, ensuring targeted localization for subsequent rsFC analysis.

### Statistical analysis

#### Descriptive statistics

The demographics and clinical continuous data are expressed as means ± standard deviations (SD). Two-tailed independent t-tests were performed to compare variables between groups when a normality assumption was held, according to a Kolmogorov-Smirnov test. Categorical data were expressed in numbers and percentages, compared by chi-square/Fisher’s exact tests. To evaluate the intervention’s effect on graph theory measures, a two-way mixed design ANOVA model was used to compare post-treatment and pre-treatment data. Effect size was evaluated using the pretest-posttest-control design (PPC) method^81^. We also used the non-parametric Mann–Whitney U test to compare group differences for minimizing possible outlier effects. To assess relationships between variables, normality was evaluated using the Shapiro–Wilk test. Pearson correlation was used to assess associations between normally distributed variables, while Spearman's rank correlation was applied for variables that did not meet normality assumptions. Uncertainty was estimated using bootstrapped 95% confidence intervals (10,000 resamples, percentile method). To address potential regression to the mean effects inherent in change score analyses, post-intervention MDS-UPDRS Part III was additionally modeled as a function of baseline MDS-UPDRS Part III and spatial performance score using linear regression as follows:

1 $${{UPDRS}3}_{{post}}={\beta }_{0}+{\beta }_{1}* {{UPDRS}3}_{{pre}}+{\beta }_{2}* \left({Spatial}\,{Performance}\,{Score}\right)+\epsilon$$

where β denotes regression coefficients and ε the error term.

To examine the relationship between neuroimaging alterations, and motor outcomes while controlling baseline differences and group, a multiple linear regression model was fitted (MATLAB’s fitlm function) as follows:

2 $${{UPDRS}3}_{{post}}={\beta }_{0}+{\beta }_{1}* {({UPDRS}3}_{{pre}})+{\beta }_{2}* \left(\triangle {\rm{MRI}}\right)+{\beta }_{3}* ({Group})+{\beta }_{4}(\triangle {MRI\; X\; Group})+\epsilon$$

where MRI denotes FA or rsFC, and Group was coded as 1 for placebo and 0 for DopApp™. Data analysis was performed using the MATLAB R2021b (MathWorks, Natick, MA) statistics and machine learning toolbox.

#### Imaging statistical analysis

The voxel-based structural connectivity statistical group analysis was performed on the quantitative maps (FA, MD, AD, RD) using a two-way mixed design ANOVA model to test the main interaction effect between time and group. A spherical region of interest with a 20 mm radius was created and centered on the peak coordinate to perform small volume correction (SVC) using false discovery rate (FDR) corrections. Data analysis was implemented in SPM12 software (http://www.fil.ion.ucl.ac.uk/spm).

At the group level, rsFC individual maps were analyzed using a two-way mixed design ANOVA model to test the main interaction effect between time and group. RsFC was considered significant at joint-probability thresholds of 0.001 at the voxel level, and *p* < 0.05 FDR corrected for multiple comparisons across the whole brain at the cluster level, with a minimum cluster size of 50 voxels. A bivariate group-level regression analysis with non-imaging covariates (e.g., psychological data, MDS-UPDRS) covariate model was used to identify global brain correlations. The analysis was implemented in SPM12 software (http://www.fil.ion.ucl.ac.uk/spm) with a parametric analysis approach across the entire brain volume^79^. The Pearson correlation was then used to test for associations with the non-imaging covariates, where the REX toolbox was used to extract cluster connectivity values^79^.

#### Sensitivity power analysis

To assess the sensitivity of the study given the small sample size, a post-hoc sensitivity power analysis was conducted using G*Power (version 3.1.9.7), assuming α = 0.05 and statistical power (1–β) = 0.80. In addition, a prospective power analysis was performed to estimate the sample size required for future studies based on the observed group means and standard deviations observed in the FA analysis. Given that voxel-based analysis was performed at the cluster level, statistical inference was corrected for multiple comparisons across spatially distinct clusters within the white matter region. In the present analysis, one significant cluster was identified; however, considering the broader search space, approximately ten spatially distinct clusters can be reasonably assumed within the analyzed white matter region. Therefore, a more conservative cluster-level multiple-comparisons Bonferroni adjustment (α = 0.005) was additionally considered to account for FDR correction across clusters.

## Supplementary information

Supplementary information

## Data Availability

The datasets generated and analyzed during the current study are not publicly available due to participant privacy and confidentiality restrictions but are available from the corresponding author on reasonable request.
