# Supplementary material for: Local and global microstructural and functional thalamomotor connectivity alterations in Parkinson’s disease following motor learning
Source: NPJ Parkinsons Dis. 2026 Jun 26;12:177. doi: 10.1038/s41531-026-01450-4 (PMC13402335; doi:10.1038/s41531-026-01450-4)
Supplement: Supplementary file 1 — Supplementary information [file 41531_2026_1450_MOESM1_ESM.pdf]

Local and global microstructural and functional thalamomotor connectivity alterations in Parkinson's disease following motor learning

Supplementary Information

Table of Content

TABLE OF CONTENT **1**

1. ELIGIBILITY CRITERIA **2**

1.1 INCLUSION CRITERIA 2
1.2 EXCLUSION CRITERIA 2
1.3 ENTRY CRITERIA/DIAGNOSIS FOR MRI ASSESSMENT 3
1.4 PROHIBITED MEDICATIONS AND TREATMENTS 3

2. THE DOPAPP INTERVENTION PROTOCOL **4**

3. STUDY OUTCOMES **7**

4. SUPPLEMENTARY TABLES **9**

TABLE S1: LINEAR REGRESSION MODEL RESULTS (SPATIAL PERFORMANCE SCORE) 9
TABLE S2: LINEAR REGRESSION MODEL RESULTS (DTI) 9
TABLE S3: LINEAR REGRESSION MODEL RESULTS (DTI) 9
TABLE S4: LINEAR REGRESSION MODEL RESULTS (RSFC) 10
TABLE S5: TOPOLOGICAL MEASURE CHANGES ANOVA (GROUP-BY-TIME) 11
TABLE S6: MATHEMATICAL DEFINITIONS OF STRUCTURAL CONNECTIVITY MEASURES 13

5. SUPPLEMENTARY FIGURES **14**

FIG. S1: STUDY FLOWCHART. 14
FIG. S2: ENHANCEMENT IN THE THALAMOMOTOR CORTEX CIRCUIT FUNCTIONAL CONNECTIVITY. ... 15
FIG. S3: DOPAPP™ SAMPLE SCREENS. 16

6. ACKNOWLEDGEMENTS **17**

7. REFERENCES **18**

1. Eligibility Criteria

1.1 Inclusion criteria

For subjects to be eligible to participate in this trial, all the following criteria must be met:

1. Male or female subjects, aged 45-80.
2. Proficient in Hebrew reading and listening comprehension (the DopApp user interface is in Hebrew).
3. Confirmed diagnosis (by a letter from treating physician) of idiopathic Parkinson's disease (PD).
4. UPDRS section 3.4 (Finger tapping) score ≥1 OR UPDRS section 3.1 (Speech) score ≥1.
5. Preserved activities of daily living: Study subjects should be able to walk, sit and stand without assistance, and own and be able to use their smartphone. They are additionally not wheelchair-bound and have good hearing and vision, naturally or corrected.
6. The study subjects are treated with levodopa containing medication (either immediate or controlled release), at a total daily dose of between 150mg and 1500mg, taken a maximum of five daily administrations. The study subject's PD drug treatment dosing regimen has been stable for the last 30 days before the study entry date and is not projected to change during the study period.
7. The subjects are able and willing to meet all protocol requirements, present themselves at the study site for all study visits and perform all tasks and tests included in the study protocol.

1.2 Exclusion criteria

A subject will be excluded from the trial for any of the following circumstances:

1. Known to have another concurrent and active neurological disease, or is known to have a significant neurological disease other than PD that may affect motor function and cognition. Examples include, but are not limited to: multiple sclerosis, epilepsy, Huntington's disease, amyotrophic lateral sclerosis (ALS), cerebral palsy, and stroke.
2. The participant is using Duopa/Duodopa, has a history of brain surgery, deep brain stimulation, a significant head injury causing loss of consciousness greater than 30 minutes, or hospitalization due to a brain injury.
3. Participants have a known history of a clinically significant intracranial abnormality (e.g., stroke, hemorrhage, space-occupying lesion) that could account for the observed symptoms (excluding abnormalities consistent with underlying PD)
4. Previously undergone focused brain irradiation for any reason.
5. Participant has a history of suicidality in the last year.
6. Known, previously treated or untreated, and/or active central nervous system (CNS) metastases and/or carcinomatous meningitis.
7. Any relevant medical, surgical, or psychiatric condition, laboratory value, or concomitant medication which makes the subject unsuitable for study entry or potentially unable to complete all aspects of the study under the investigator's discretion.
8. Has regularly used opioids, which include, but are not limited to morphine, codeine, oxycodone, hydrocodone, fentanyl, tramadol, and methadone, more than three times per week in the 15 days prior to enrollment.
9. At screening, is a known regular user (including "recreational use") of any illicit drugs or has a history within the last year of active substance abuse including (but not limited to): alcohol, cocaine, crack cocaine, ecstasy, hallucinogens (such as LSD, PCP, mushrooms, and salvia), heroin, inhalants (such as spray paints, markers, cleaning supplies), ketamine, meth, etc.

10. Known to have a neoplastic disorder, which is either currently active or has been in remission for less than one year.
11. Pregnant
12. Inability to sign the informed consent form.
13. Subject has participated in another study of an investigational medicinal product (IMP) or a medical device within the last 30 days or is currently participating in another study of an IMP or medical device.
14. Does not own a smartphone.

1.3 Entry criteria/diagnosis for MRI assessment

1. Subject age is ≤76
2. Right-handed only
3. The subject is able and consents to undergo MRI scans.
4. The subject experiences dyskinesia of the head and neck less than 50% of the day.
5. Does not have a condition which is contra-indicated with MRI (metal implants, claustrophobia etc. according to the MRI safety form).

1.4 Prohibited medications and treatments

Subjects are not eligible to participate in this study if they received the following treatments within 30 days prior to entry into the study (Study Visit 1a), unless differently specified. In addition, these medications are prohibited to be administered to study subjects also during the conduct of the study. If treatment with these medications/therapies must be initiated after the subject has already entered the study, the subject should be withdrawn from the study ASAP:

1. Chronic treatment with anti-depressants, including SSRIs, SNRIs, NDRIs, and TCAs is not allowed if their type and/or dosing regimen has been initiated and/or changed within a period of 30 days prior to study entry. It is also not permitted to change the type and/or dosing regimen of these drugs during the trial.
2. Chronic or frequent (more than three times per week) use of anxiolytic drugs, such as (but not limited to) benzodiazepines, except if taken at bedtime only.
3. Frequent (more than three times per week) use of opioids, which include, but are not limited to morphine, codeine, oxycodone, hydrocodone, fentanyl, tramadol, and methadone.
4. Treatment with non-pharmacological treatments for PD included in the app (speech, etc.) for a period of 30 days prior to study entry and during the trial.
5. Use of drugs such as barbiturates and St. John's wort that are known to affect metabolism of PD drugs.

2. The DopApp intervention protocol

Each daily self-training session was originally designed to last approximately 30 minutes (excluding walking) and is comprised of the daily components described below. Importantly, all activities were also available on demand via a content library, allowing participants to engage in additional training beyond the standard daily protocol.

DAY	NAME OF INTERVENTION	EXPLANATION
ALL	Each day, the activities included the following: maze game (a multisensory, gradual visual masking spatial navigation game), amplitude game (part of speech therapy, designed to train increasing loudness), pitch game, (part of speech therapy, designed to train pitch range), typing game (part of fine-motor training), reading game (part of speech therapy, design to train improved clarity of speech), whack-a-mole game (part of fine-motor training, the participant has to quickly respond to appearing targets and repress response to distractors), Simon game (a game combining fine motor and auditory memory training, where the participant has to recall a sequence of sounds and enter it) and walking encouragement (participants were reminded to walk for at least 15 minutes daily).	
1	**Program introduction**	A brief introduction of the program
	**Setting medication reminders**	
	**Walking activity**	Set up
	**Speech therapy introduction**	An overview of the purpose of speech therapy.
	**Vocal warm-up**	Explanation
	**Setting volume target intensity**	
	**Amplitude**	Tutorial and game
	**Vocal range**	
	**Pitch**	Tutorial and game
	**Explanation of reading practice**	
	**Reading**	Tutorial and game
	**Maze**	Tutorial and game
	**Introduction of fine motor skills**	Describe how fine motor skills contribute to various tasks and activities.
	**Typing**	Tutorial and game
	**Whack**	Tutorial and game
	**Simon**	Tutorial and game
2	**About the app**	A brief explanation of the application's daily operating schedule and the planned activities.
	**Muscle relaxation**	A brief video outlines the concept of progressive muscle relaxation.
	**Muscle relaxation practice**	Audio
	**effective vocal practice**	A brief video demonstrating how to practice voice and speech training.
	**Toilet paper folding practice**	A fine motor exercise.
3	**Traditional medicine and the app**	A brief video explaining the relationship between traditional medicine and the app.
	**Guided imagery**	A brief video introducing and explaining guided imagery as a therapeutic tool.
	**Guided imagery to a safe place**	Audio
	**Neuroplasticity**	A brief video about neuroplasticity.
4	**Motivation**	A brief video describing how the disease impacts the motivational system, the ability to take initiative, follow through with beneficial actions, and maintain engagement.
	**Guided imagery for relaxation**	Audio

	**Multi-senses**	A brief video about multi-senses.
	**Tips for optimal movement**	A video describing three tips for optimal movement.
**5**	Coping with stress and anxiety	
	**Breathing practice explanation**	A brief video demonstrating how breathing exercises can be used to manage and reduce stress.
	**About diaphragmatic breathing**	
	**Practice diaphragmatic breathing**	Interactive breathing video
	**Coordination practice**	Alternating number marking
	**About Neurodance**	A video demonstrating how dance can serve as a therapeutic tool.
	**Neurodance - fast paced movement**	
**6**	**Guided imagery for medication intake**	Explanation and practice
	**Mindful eating**	Audio
	**Practicing fine motor skills**	Ball exercise
**7**	**Week 1 Summary**	
	**Box breathing interactive breathing**	Interactive activity
	**Coordination**	Finger movement sequences
8	**Body scan**	A brief video explaining a technique for directing our attention to different body parts, proven to enhance sleep, memory, and more.
	**Body scan practice**	Audio
	**Writing exercise**	explanation and practice
**9**	**Thoughts and their interpretation**	Explain how our thoughts influence our perception of reality and behavior, and how becoming aware of them can improve our wellbeing.
	**Activity choices**	Progressive muscle relaxation/diaphragmatic breathing/guided imagery to a safe place.
	**Coordination**	Movement sequences with rhythm
**10**	**Cognitive biases**	A video explaining cognitive biases.
	**Activity choices**	Body scan/box breathing/guided imagery for relaxation
	**Toilet paper folding practice**	A fine motor exercise
	**Physiotherapy - stretches**	Improving motor skills.
**11**	**Balanced thoughts explanation**	An explanation of the steps to take after recognizing an extreme thought.
	**Balanced thoughts activity**	Interactive activity
	**Physiological sigh practice**	A brief introduction with a new breathing technique based on diaphragmatic breathing.
	**Activity choices**	Box breathing/diaphragmatic breathing practice.
	**Fine motor skills**	Practicing with a pen.
	**Physiotherapy**	Motor skills practice 1
**12**	**Breathing awareness**	Explanation and practice
	**Alternating number marking**	
	**Neuro-dance - balance**	

**13**	**Parable of the two arrows**	A short video explaining the parable of the arrows, which explores whether we experience less suffering in response to life's challenges.<br>Body scan/breathing awareness practice.
	**Practicing fine motor skills**	Ball exercise
**14**	**Week 2 summary**	
	**White bear effect**	Video
	**Activity choices**	Diaphragmatic breathing/guided imagery for relaxation/guided imagery for medication intake practice.
	**Coordination**	Finger movement sequences
	**Physiotherapy**	Motor skills practice 2
**15**	**Struggle switch**	A short video explaining the "struggle switch" – our natural response to unpleasant emotions is to resist, but accepting these feelings can help reduce their impact.
	**Activity choices**	Body scan/box breathing/progressive muscle relaxation.
	**Writing exercise**	Fine motor exercise.
	**Physiotherapy**	Hand stretches
**16**	**Collecting positive experiences**	A short video about collecting positive experiences: Exploring why we tend to focus on negative events and how we can shift our attention to positive ones.
	**Activity choices**	Guided imagery to a safe place/body scan/breathing awareness practice.
	**Coordination**	Movement sequences with rhythm.
	**Physiotherapy**	Motor skills practice 3.
**17**	**Activity choices**	Body scan/box breathing/progressive muscle relaxation practice.
	**Flexibility and adaptability**	Video
	**Physiotherapy**	Neck stretches
**18**	**Worry cycle**	A video explaining the psychosomatic cycle: how extreme interpretations of unpleasant feelings can intensify stress and anxiety, along with techniques to break free from this cycle.
	**Activity choices**	Progressive muscle relaxation/breathing awareness /guided imagery to a safe place.
	**Toilet paper folding practice**	Video
	**Physiotherapy**	Opening and closing the palms.
**19**	**Avoidance and struggle**	A short video on avoidance and struggle. Explains that while avoiding or struggling with unpleasant feelings may provide short-term relief, it is ultimately ineffective in the long term.
	**Activity choices**	Guided imagery for medication intake/box.<br>Breathing/body scan
	**Fine motor skills**	Practicing with a pen
	**Neuro-dance**	Wide and open movements
**20**	Acceptance	A short video on acceptance: an essential aspect of emotional regulation and coping with difficult situations is the ability to accept the current reality as it is. This approach helps us navigate challenges without surrendering to them.
	**Activity choices**	Body scan/breathing awareness/guided imagery practice.
	**Coordination**	Finger movement sequences
	**Physiotherapy**	Getting up from a chair
**21**	**Practicing fine motor skills**	Ball exercise
	**Program summary**	

3. Study Outcomes

Primary Outcome

MDS-UPDRS total scores (sum of parts I, II & III): The inter-group difference in the mean change from baseline (Visit 1) to the end of the study period (Visit 2) in the sum of the MDS-UPDRS score for the sum of parts I, II & III.

Secondary Outcomes

**MDS-UPDRS Assessments**[1]	
**Parts** I-IV	MDS-UPDRS score sum of parts I, II, III & IV: the inter-group difference in the mean change from baseline (Visit 1) to the end of the study period (Visit 2) in the MDS-UPDRS score for sum of parts I, II, III & IV.
**Individual parts**	MDS-UPDRS score for each of parts I, II, III & IV individually: the inter-group difference in the mean change from baseline (Visit 1) to the end of the study period (Visit 2) in the MDS-UPDRS score in each part (I-IV) separately.
**Parts** II & III	MDS-UPDRS score sum of parts II & III: The inter-group difference in the mean change from baseline (Visit 1) to the end of the study period (Visit 2) in the MDS-UPDRS score for sum of parts II & III.
**Parts** I & II	MDS-UPDRS score sum of parts I & II: The inter-group difference in the mean change from baseline (Visit 1) to the end of the study period (Visit 2) in the MDS-UPDRS score for sum of parts I & II.
**Speech**	MDS-UPDRS sections related to speech and oral muscle function total = MDS-UPDRS Speech (#2.1; #2.2; #2.3; #2.4; #3.1; #3.2): The inter-group difference in the mean change from baseline (Visit 1) to the end of the study period (Visit 2) in the MDS-UPDRS speech sections listed above.
**Fine hand motor**	MDS-UPDRS related to fine motor skills and hand movement total = MDS-UPDRS fine hand motor (#2.4; #2.5, #2.6, #2.7; #2.8, #3.4; #3.5, #3.6 #3.15, #3.16). The inter-group difference in the mean change from baseline (Visit 1) to the end of the study period (Visit 2) in the MDS-UPDRS fine hand motor sections listed above.
**Proportion of Responders by MDS-UPDRS Improvement**	The inter-group difference in the proportion of subjects in each group, achieving predefined levels of change in the MDS-UPDRS total score from baseline (Visit 1) to the end of the study period (Visit 2). Specifically, we assessed the proportion of subjects reaching at least the following thresholds:<br>o A reduction of at least 5.5 points ("Minimal Detectable Response Rate").<br>o A reduction of at least 6.7 points ("Minimal (MCID) Response Rate").<br>o A reduction of at least 10 points.<br>o A reduction of at least 20% in the score compared to baseline visit score.

	○ A reduction of at least 30% in the score compared to baseline visit score.
**PD Clinical Outcomes**	
**PDQ-39**[2]	The inter-group difference in the mean change from baseline (Visit 1) to the end of the study period (Visit 2) in PDQ-39. A summary index is then calculated as the sum of the total score of the dimensions divided by the number of dimensions, i.e. sum of dimension scores /8. If any item score is missing, the relevant dimension score and the summary index will be missing.
**VHI-HEB**[3]	The inter-group difference in the mean change from baseline (Visit 1) to the end of the study period (Visit 2) in VHI total score and in each subscale (functional/physical/emotional) separately.
**PDSS-2**[4]	The inter-group difference in the mean change from baseline to the end of the study period (Visit 2) in the PDSS score.
**Psychological & Cognitive Outcomes**	
**TMT A&B**[5]	The inter-group difference in the mean change from baseline (Visit 1) to the end of the study period (Visit 2) in TMT A & B total score and in each of them separately.
**BDI-II**[6]	The inter-group difference in the mean change from baseline (Visit 1) to the end of the study period (Visit 2) in the BDI-II total score.
**STAIT-5/ STAIS-5**[7]	The inter-group difference in the mean change from baseline (Visit 1) to the end of the study period (Visit 2) in the STAIT-5/STAIS-5 total score.
**BRCS**[8]	The inter-group difference in the mean change from baseline (Visit 1) to the end of the study period (Visit 2) in the BRCS total score.
**PSS-10**[9]	The inter-group difference in the mean change from baseline (Visit 1) to the end of the study period (Visit 2) in the PSS-10 total score.
**NHC-SF**[10]	The inter-group difference in the mean change from baseline (Visit 1) to the end of the study period (Visit 2) in the MHC-SF total score.
**Brain Imaging Outcomes**	
**Resting state fMRI**	Inter-group resting state fMRI connectivity changes from baseline (Visit 1) to the end of the study period (Visit 2) (peak connectivity coordinates, significance value, and cluster size). Contrast: DopApp arm vs. placebo arm.
**DTI**	Inter-group diffusion tensor imaging (DTI) changes from baseline (Visit 1) to the end of the study period (Visit 2): quantitative evaluation of microstructure changes in grey and white matter based on the fractional anisotropy (FA), mean diffusivity (MD), and the parallel and radial diffusivities maps, and for white matter structural connectivity determination. Additionally, alterations in topological measures (Betweenness Centrality, Efficiency, Strength) is evaluated.
**DopApp use Outcomes**	
App Engagement	Measuring app usage and activity levels. App engagement is calculated for the trial group as number of days the users completed their daily digital treatment protocol during trial period.
**Safety Outcomes**	
Adverse events	Adverse events.

4. Supplementary Tables

Table S1: Linear regression model results (Spatial Performance Score)

	Predictor	β	SE	t	p
β0	Intercept	-3.368	4.596	-0.733	0.478
β1	Baseline MDS-UPDRS Part III	1.073	0.173	6.203	<0.001
β2	Spatial Performance Score	-4.397	1.862	-2.361	0.036

β = Beta coefficient (Estimate), SE = Standard Error, t = t-statistic, p = p-value

Table S2: Linear regression model results (DTI)

	Predictor	β	SE	t	p
β0	Intercept	18.555	1.453	12.770	**<0.001**
β1	Baseline MDS-UPDRS Part III	10.971	1.026	10.693	**<0.001**
β2	delta FA @ VLp*	-3.967	1.705	-2.327	**0.028**
β3	Group	2.231	2.446	0.912	0.370
β4	delta FA X Group	1.975	1.554	1.271	0.214

* Refers to the DopApp group. Group was coded as 1 for placebo and 0 for DopApp. β = Beta coefficient (Estimate), SE = Standard Error, t = t-statistic, p = p-value

Table S3: Linear regression model results (DTI)

	Predictor	β	SE	t	p
β0	Intercept	8.239	0.837	9.846	**<0.001**
β1	Baseline MDS-UPDRS Hand motor	4.110	0.607	6.769	**<0.001**
β2	delta FA @ VLp*	-2.682	0.984	-2.727	**0.011**
β3	Group	0.096	1.409	0.068	0.946
β4	delta FA X Group	1.707	0.913	1.871	0.072

* Refers to the DopApp group. Group was coded as 1 for placebo and 0 for DopApp. β = Beta coefficient (Estimate), SE = Standard Error, t = t-statistic, p = p-value

Table S4: Linear regression model results (rsFC)

	Predictor	β	SE	t	p
β0	Intercept	22.728	1.734	13.109	<0.001
β1	Baseline MDS-UPDRS Part III	10.739	1.117	9.614	<0.001
β2	delta rsFC @ VLp-PreCG	-1.662	2.019	-0.516	0.610
β3	Group	-6.387	2.483	-2.573	0.016
β4	delta rsFC X Group	-0.618	1.876	-0.330	0.744

* Refers to the DopApp group. Group was coded as 1 for placebo and 0 for DopApp. β = Beta coefficient (Estimate), SE = Standard Error, t = t-statistic, p = p-value

Table S5: Topological measure changes ANOVA (group-by-time)

	P-value Baseline	Effect size*	Main effect of group		Main effect of time		Interaction effect	
			F	P-value	F	P-value	F	P-value
**Local Node (n) = right PreCG**								
**Graph weights: FA**								
Global Density	0.598	0.421	0.748	0.394	0.003	0.959	1.393	0.247
Global Efficacy	0.898	0.091	0.083	0.776	0.084	0.774	0.066	0.800
Mean Local Efficacy	0.587	0.120	0.528	0.473	0.020	0.889	0.114	0.738
Global BC	0.433	-0.081	1.029	0.319	0.006	0.938	0.052	0.821
Local Efficacy (n)	0.396	0.755	0.008	0.930	1.016	0.321	4.493	**0.042**
BC (n)	0.865	-0.244	0.188	0.667	0.432	0.516	0.467	0.499
Strength (n)	0.731	0.253	0.010	0.923	0.001	0.976	0.503	0.484
**Graph weights: average tract length**								
Global Density	0.598	0.421	0.748	0.394	0.003	0.959	1.393	0.247
Global Efficacy	0.978	0.550	0.759	0.391	0.600	0.445	2.385	0.133
Mean Local Efficacy	0.611	0.515	1.721	0.200	1.356	0.253	2.093	0.158
Global BC	0.520	-0.103	0.798	0.379	0.035	0.853	0.084	0.774
Local Efficacy (n)	0.357	0.874	0.184	0.671	0.801	0.378	6.017	**0.020**
BC (n)	0.809	-0.070	0.025	0.875	0.698	0.410	0.038	0.846
Strength (n)	0.626	0.318	0.008	0.930	0.486	0.491	0.799	0.379
**Graph weights: number of tracts**								
Global Density	0.598	0.421	0.748	0.394	0.003	0.959	1.393	0.247
Global Efficacy	0.273	0.424	0.509	0.481	0.014	0.906	1.415	0.244
Mean Local Efficacy	0.387	0.421	0.291	0.594	0.010	0.920	1.397	0.247
Global BC	0.940	-0.349	0.251	0.620	0.135	0.716	0.957	0.336
Local Efficacy (n)	0.277	0.767	0.011	0.917	0.424	0.520	4.638	**0.039**
BC (n)	0.881	-0.001	0.057	0.812	0.273	0.605	0.000	0.999
Strength (n)	0.249	0.677	0.206	0.654	0.420	0.522	3.610	0.067
**Graph weights: MD**								
Global Density	0.598	0.421	0.748	0.394	0.003	0.959	1.393	0.247
Global Efficacy	0.804	-0.001	0.076	0.785	0.016	0.900	0.000	0.997
Mean Local Efficacy	0.542	-0.001	0.416	0.524	0.151	0.700	0.000	0.999
Global BC	0.429	-0.082	1.043	0.315	0.006	0.939	0.053	0.820
Local Efficacy (n)	0.511	0.413	0.007	0.935	0.228	0.636	1.346	0.255
BC (n)	0.264	0.121	1.215	0.279	0.703	0.409	0.115	0.737
Strength (n)	0.876	0.166	0.018	0.894	0.033	0.857	0.217	0.645

Local Node (N) = left PreCG								
**Graph weights: FA**								
Global Density	0.597	0.421	0.751	0.393	0.002	0.968	1.395	0.247
Global Efficacy	0.898	0.091	0.083	0.776	0.084	0.774	0.066	0.800
Mean Local Efficacy	0.577	0.114	0.545	0.466	0.028	0.868	0.102	0.752
Global BC	0.433	-0.081	1.029	0.319	0.006	0.938	0.052	0.821
Local Efficacy (n)	0.438	-0.382	0.088	0.769	1.790	0.191	1.151	0.292
BC (n)	0.773	0.123	0.376	0.544	4.408	0.044	0.118	0.733
Strength (n)	0.536	-0.161	0.226	0.638	0.290	0.594	0.204	0.655
**Graph weights: average tract length**								
Global Density	0.597	0.421	0.751	0.393	0.002	0.968	1.395	0.247
Global Efficacy	0.507	0.512	1.572	0.220	3.779	0.061	2.064	0.161
Mean Local Efficacy	0.605	0.511	1.737	0.197	1.378	0.250	2.059	0.162
Global BC	0.520	-0.103	0.798	0.379	0.035	0.853	0.084	0.774
Local Efficacy (n)	0.706	0.013	0.239	0.629	0.014	0.905	0.001	0.972
BC (n)	0.488	0.043	0.627	0.435	0.005	0.942	0.014	0.905
Strength (n)	0.853	-0.056	0.097	0.758	3.134	0.087	0.024	0.877
**Graph weights: number of tracts**								
Global Density	0.597	0.421	0.751	0.393	0.002	0.968	1.395	0.247
Global Efficacy	0.273	0.424	0.509	0.481	0.014	0.906	1.415	0.244
Mean Local Efficacy	0.384	0.427	0.292	0.593	0.013	0.908	1.437	0.240
Global BC	0.940	-0.349	0.251	0.620	0.135	0.716	0.957	0.336
Local Efficacy (n)	0.483	-0.082	0.658	0.424	0.252	0.619	0.053	0.819
BC (n)	0.358	-0.282	0.541	0.468	1.252	0.272	0.625	0.435
Strength (n)	0.270	0.686	0.252	0.619	1.129	0.296	3.709	0.064
**Graph weights: MD**								
Global Density	0.597	0.421	0.751	0.393	0.002	0.968	1.395	0.247
Global Efficacy	0.804	-0.001	0.076	0.785	0.016	0.900	0.000	0.997
Mean Local Efficacy	0.535	-0.005	0.425	0.519	0.163	0.689	0.000	0.990
Global BC	0.429	-0.082	1.043	0.315	0.006	0.939	0.053	0.820
Local Efficacy (n)	0.354	-0.124	0.340	0.564	0.293	0.592	0.121	0.730
BC (n)	0.945	0.084	0.053	0.820	0.821	0.372	0.056	0.815
Strength (n)	0.303	-0.244	0.649	0.427	0.527	0.474	0.469	0.499

* pretest-posttest-control design (PPC) method, PreCG, precentral gyrus

Table S6: Mathematical definitions of structural connectivity measures

Measure	Definition	Equation
**Mean Diffusivity**	The average of all three eigenvalues (The overall magnitude of diffusion)	$MD = \langle\lambda\rangle = \frac{1}{3}\sum_{i=1}^{3}\lambda_i; \quad i = 1..3$
**Fractional Anisotropy**	The normalized variance of the three eigenvalues about their mean, and quantifies the directional diffusion	$FA = \sqrt{\frac{3}{2}}\frac{\sqrt{\sum_{i=1}^{3}(\lambda_i - \langle\lambda\rangle)^2}}{\sqrt{\sum_{i=1}^{3}\lambda_i^2}}; \quad i = 1..3$
**Radial Diffusivity**	The perpendicular water diffusion to the principal axis	$RD = \frac{1}{2}(\lambda_2 + \lambda_3)$
**Axial Diffusivity**	Water diffusion along the principal axis	$AD = \lambda_1$
**Density weight**	Density is the fraction of present edges to possible connections	$D = \frac{2E}{N(N-1)}$
**Global Efficacy**	Average of inverse shortest path length in the entire network.	$E = \frac{1}{n}\sum_{i\in N}\frac{\sum_{j\in N, j\neq i} d_{ij}^{-1}}{n-1}$
**Degree of a Node**	The number of edges connected to a node (not weighted).	$k_i = \sum_{j\in N} a_{ij}$
**Strength**	The sum of weights of edges connected to a node	$S = K_i^w = \sum_{j\in N} w_{ij}$
**Local Efficiency**	Computed on the neighborhood of the node (i.e. the average of inverse shortest path length from node i to its first-degree neighbors).	$E_{loc} = \frac{1}{2}\sum_{i\in N}\frac{\sum_{j,h\in N, j\neq i}(w_{ij}w_{ih}[d_{ih}^{w}(N_i)]^{-1})^{1/3}}{k_i(k_i-1)}$
**Betweenness Centrality**	The fraction of all shortest paths in the network that pass through a given node.	$BC_i = \frac{1}{(n-1)(n-2)}\sum_{\substack{h,j\in N \\ h\neq j, h\neq i, j\neq i}}\frac{\rho_{hj(i)}}{\rho_{hj}}$ where, ρ_{hj} is the number of shortest paths between h and j, and $\rho_{hj}(i)$ is the number of shortest paths between h and j that passes through i.

where, λi, i=1..3, are the eigenvalues ($l_1 > l_2 > l_3 > 0$) of the diffusion tensor matrix. N is the set of all nodes in the network, E is the number of nonzero edges, d is the distance between nodes i and j, aij is the connection status between i and j (0/1), wij is the weight of the ij connection: weights are normalized, such that 0 ≤ wij ≤ 1 for all i and j [11-13].

5. Supplementary Figures

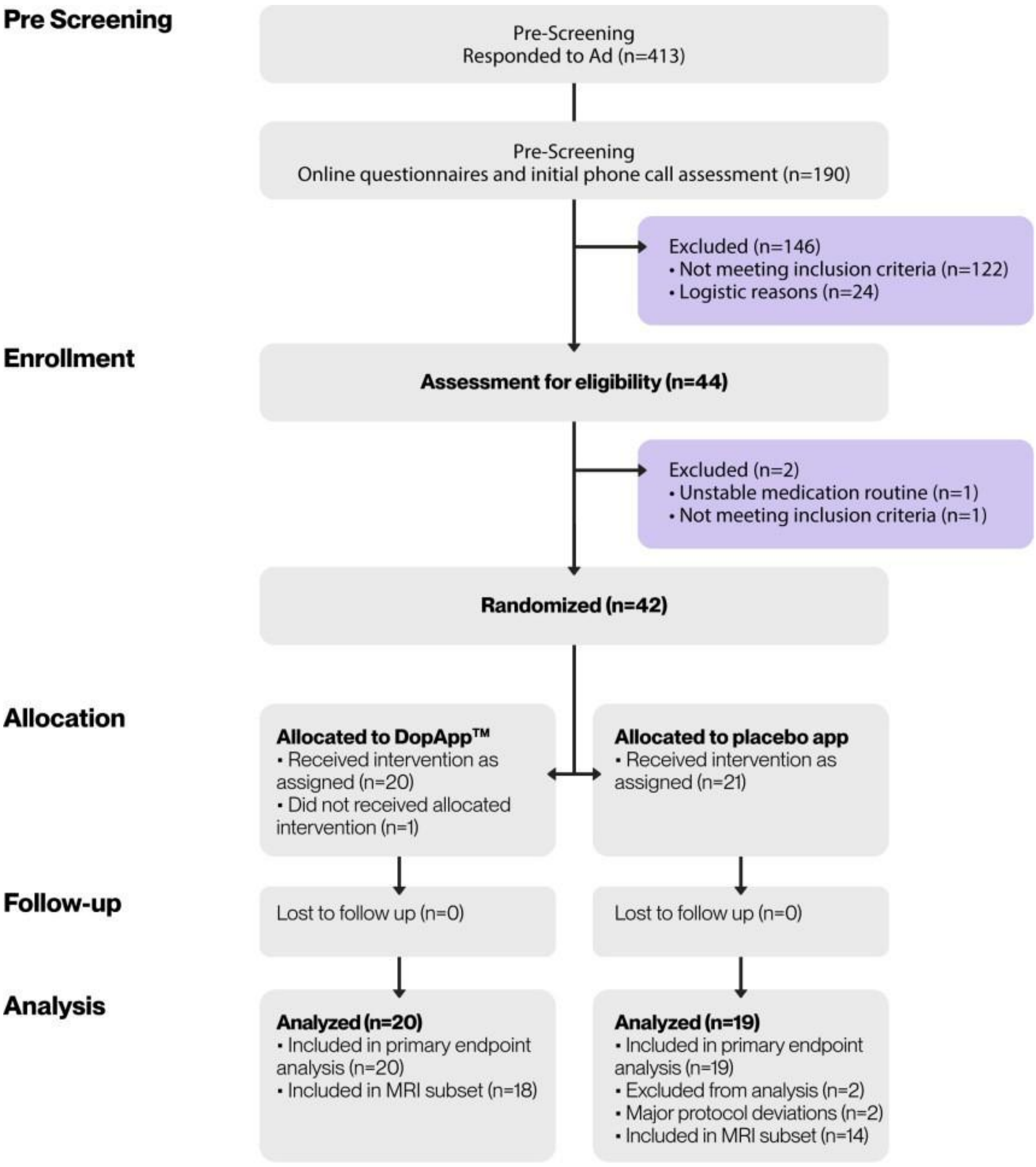

Fig. S1: Study flowchart.

A total of 413 individuals responded to the study advertisement, of whom 190 completed the prescreening questionnaire. Many were excluded for not meeting eligibility criteria or due to technical issues. Ultimately, 44 PwP were screened, 42 met the inclusion criteria and were enrolled and randomized. One participant in the DopApp™ group withdrew consent prematurely, and two in the placebo group were excluded due to major protocol deviations. A total of 39 participants completed the study per protocol. Of them, 32 were included in the MRI analysis.

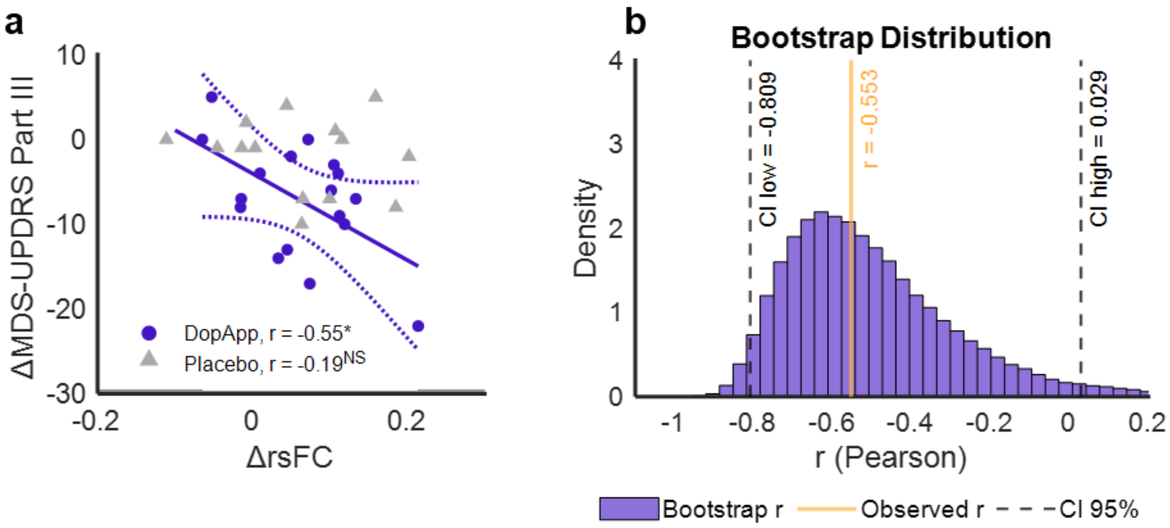

Fig. S2: Enhancement in the thalamomotor cortex circuit functional connectivity.

(a) The scatter plot depicts the relationship between VLp-PreCG rsFC changes and clinical motor symptom improvements, as assessed by MDS-UPDRS Part III. **(b)** Bootstrap distribution of the Pearson correlation coefficient between spatial performance score and change in MDS-UPDRS Part III. The histogram represents the normalized distribution of Pearson r values obtained from 10,000 bootstrap resamples. No significant associations were found in the placebo group. r, Pearson correlations value, purple dashed lines, 95% prediction bounds, CI, confidence intervals, *P<0.05, VLp: ventral lateral posterior, PreCG: precentral gyrus.

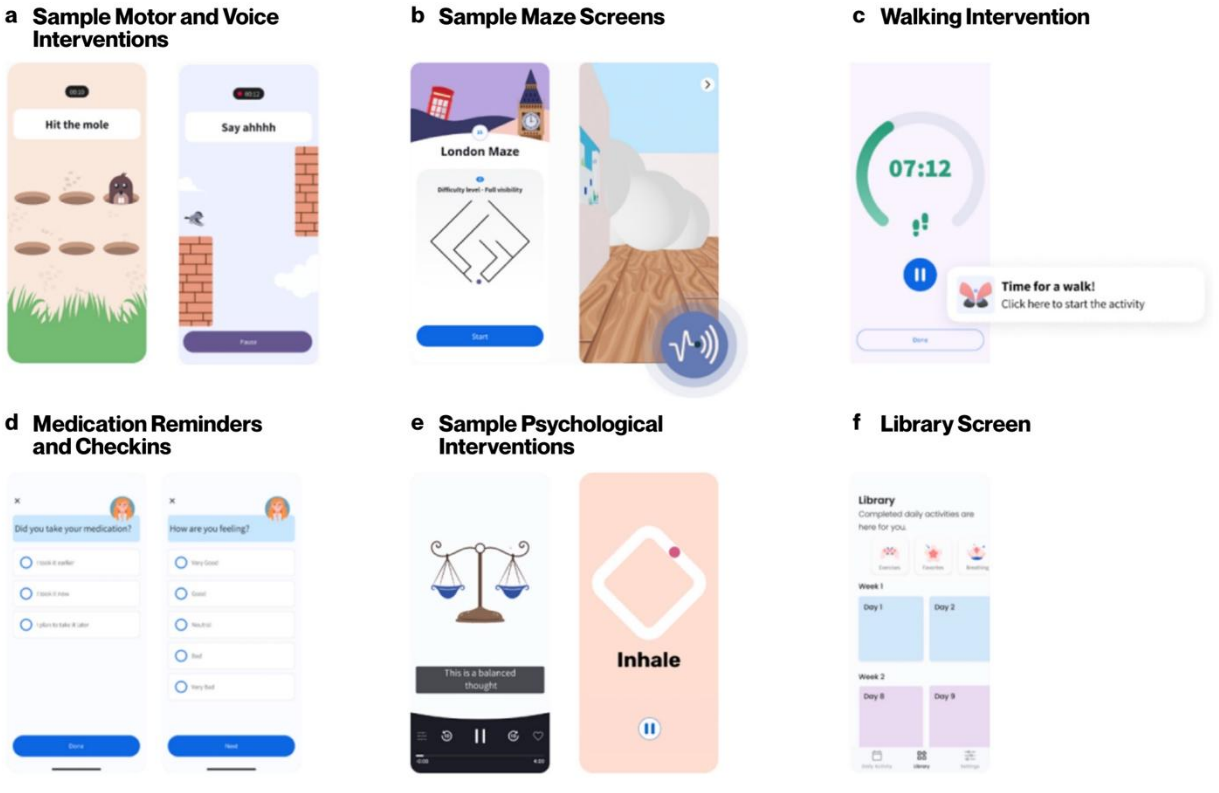

Fig. S3: DopApp™ Sample Screens.

(a) Sample motor and voice interventions: fine motor game of hit-the-mole (left) and pitch modulation training game (right). (**b)** Sample sensorimotor-deprivation spatial memory screens: The exercise features virtual spatial navigation exercises that utilize digital 3D Hebb-Williams mazes and incorporates both allocentric and egocentric navigation techniques. This exercise is implemented through a progressive, three-step vision-deprivation sensorimotor training process, designed to increase navigation complexity over time. Each maze trial starts with a top-down 2D map view for allocentric navigation (left). Participants then navigate the maze fully sighted, using auditory cues for spatial information, such as distance from walls. After completing this phase, the 2D map is shown again, followed by a more challenging 3D navigation phase where 50% of the maze is masked (right). In the final phase, participants navigate blindfolded using only auditory feedback, employing a sensory substitution strategy. This approach aims to enhance brain connectivity, spatial learning, and balance across sensory and cognitive networks[14]. **(c)** Walking intervention: reminder (right) and walking timer. (**d**) Medication reminder (left) and check-in (right). (**e**) Sample psychological interventions: CBT module on balanced thoughts (left) and breathing exercise (right). (**f**) Library screen. A detailed explanation of the intervention protocol is provided in the **Supplementary Information**.

6. Acknowledgements

We would like to thank Dr. Shira Molcho, Dr. Sheila Oren, and Dr. Shai Erlich for their medical supervision and clinical support. With deep gratitude, we recognize the special contribution of Gal Gorelick and Shai Gorelick, the study coordinators, Dina Kofler, the study CRA, Lior Benderski, Hagar Tamir, Hadas Arzi, Gal Yogev and Maya Goldberger, the installation and onboarding team, Bar Grisaru, the technical support representatives, Ofek Salama, the Brain, Cognition & Technology Lab operations manager, Dalit Shlayn, the MRI technologist, and Dr. Dikla Ender-Fox, director of the Ruth and Meir Rosental Brain Imaging Center, without whom this study would not have been possible. We also thank Eran Etam, Shahar Har Nesher and the DopApp™ product and development team at Remepy, for delivering a high-quality application, and the Remepy leadership team, Dr. Michal Tsur and Or Shoval for continued guidance and support. We thank Yuval Levitan, Noa Cohen, Anat Zait, Roni Peled and Ariel Shahaf for professional assistance in creating the multimodal interventions in DopApp™. We thank Dr. Mechael Kanovsky for medical writing support.

7. References

1 Goetz, C. G. *et al.* Movement Disorder Society-sponsored revision of the Unified Parkinson's Disease Rating Scale (MDS-UPDRS): scale presentation and clinimetric testing results. *Mov Disord* **23**, 2129-2170, doi:10.1002/mds.22340 (2008).

2 Jenkinson, C., Fitzpatrick, R., Peto, V., Greenhall, R. & Hyman, N. The Parkinson's Disease Questionnaire (PDQ-39): development and validation of a Parkinson's disease summary index score. *Age and ageing* **26**, 353-357 (1997).

3 Jacobson, B. H. *et al.* The voice handicap index (VHI) development and validation. *American journal of speech-language pathology* **6**, 66-70 (1997).

4 Trenkwalder, C. *et al.* Parkinson's disease sleep scale—validation of the revised version PDSS-2. *Movement Disorders* **26**, 644-652 (2011).

5 Tombaugh, T. N. Trail Making Test A and B: normative data stratified by age and education. *Archives of clinical neuropsychology* **19**, 203-214 (2004).

6 Beck, A. T., Steer, R. A. & Brown, G. Beck depression inventory–II. *Psychological assessment* (1996).

7 Zsido, A. N., Teleki, S. A., Csokasi, K., Rozsa, S. & Bandi, S. A. Development of the short version of the spielberger state—trait anxiety inventory. *Psychiatry research* **291**, 113223 (2020).

8 Sinclair, V. G. & Wallston, K. A. The development and psychometric evaluation of the Brief Resilient Coping Scale. *Assessment* **11**, 94-101 (2004).

9 Cohen, S., Kamarck, T., Mermelstein, R. J. J. o. h. & behavior, s. A global measure of perceived stress. 385-396 (1983).

10 Lamers, S. M., Westerhof, G. J., Bohlmeijer, E. T., ten Klooster, P. M. & Keyes, C. L. J. J. o. c. p. Evaluating the psychometric properties of the mental health continuum-short form (MHC-SF). **67**, 99-110 (2011).

11 Latora, V. & Marchiori, M. J. P. r. l. Efficient behavior of small-world networks. **87**, 198701 (2001).

12 Rubinov, M. & Sporns, O. J. N. Complex network measures of brain connectivity: uses and interpretations. **52**, 1059-1069 (2010).

13 Alexander, A. L., Hasan, K., Kindlmann, G., Parker, D. L. & Tsuruda, J. S. J. M. R. i. M. A. O. J. o. t. I. S. f. M. R. i. M. A geometric analysis of diffusion tensor measurements of the human brain. **44**, 283-291 (2000).

14 Amedi, A., Shelly, S., Saporta, N. & Catalogna, M. Perceptual learning and neural correlates of virtual navigation in subjective cognitive decline: A pilot study. *iScience* (2024).
